# Supplementary material for: A Transient Metabolic State in Melanoma Persister Cells Mediated by Chemotherapeutic Treatments
Source: Front Mol Biosci. 2022 Jan 27;8:780192. doi: 10.3389/fmolb.2021.780192 (PMC8829428; doi:10.3389/fmolb.2021.780192)
Supplement: Supplementary file 1 [file DataSheet1.pdf]

SUPPLEMENTARY MATERIAL

A TRANSIENT METABOLIC STATE IN MELANOMA PERSISTER CELLS MEDIATED BY CHEMOTHERAPEUTIC TREATMENTS

Prashant Karki[†], Vahideh Angardi[†], Juan C. Mier and Mehmet A. Orman*

Department of Chemical and Biomolecular Engineering, University of Houston, Houston, TX, USA

[†] These authors contributed equally.

* Corresponding Author: S222 Engineering Bldg 1, 4726 Calhoun Rd, Houston, TX 77204, Phone: 713-743-6785, Email: morman@central.uh.edu

SUPPLEMENTARY FIGURES

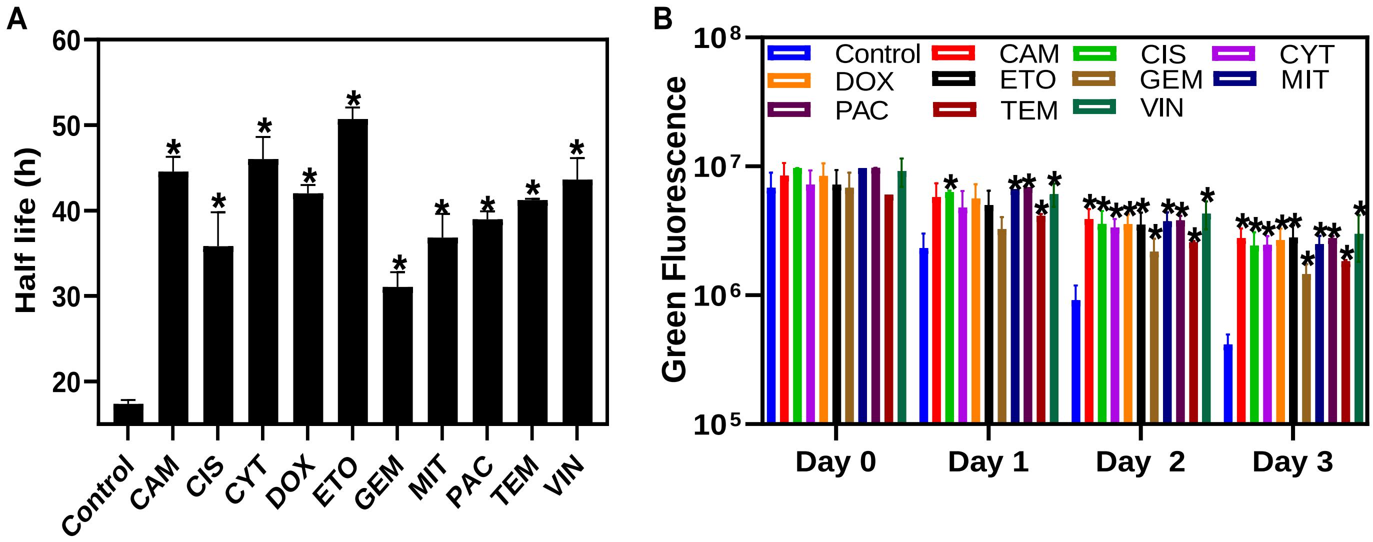

Supp. Figure S1: Chemotherapeutic agents induce cell cycle arrest. A375 melanoma cells prestained with CFSE dye were treated with indicated chemotherapeutic agents, and their fluorescence intensity was monitored for 3 days with flow cytometry. For control, cells were treated with the solvent only. The concentrations of chemotherapeutic agents were 10xIC_{50}, except TEM, whose concentration was 5xIC_{50} (Supp. Table S2). **(A)** The mean fluorescence intensity for the first 3 days for each group was integrated into the fluorescence decay equation to calculate the half-life of the fluorescent signal. * indicates a significant difference between control and treatment groups ($P<0.05$). N=3. **(B)** Mean fluorescence intensity for each group was provided by a bar graph. Each data point represents the mean value ± standard error. * indicates a significant difference between control and treatment groups ($P<0.05$). N=3.

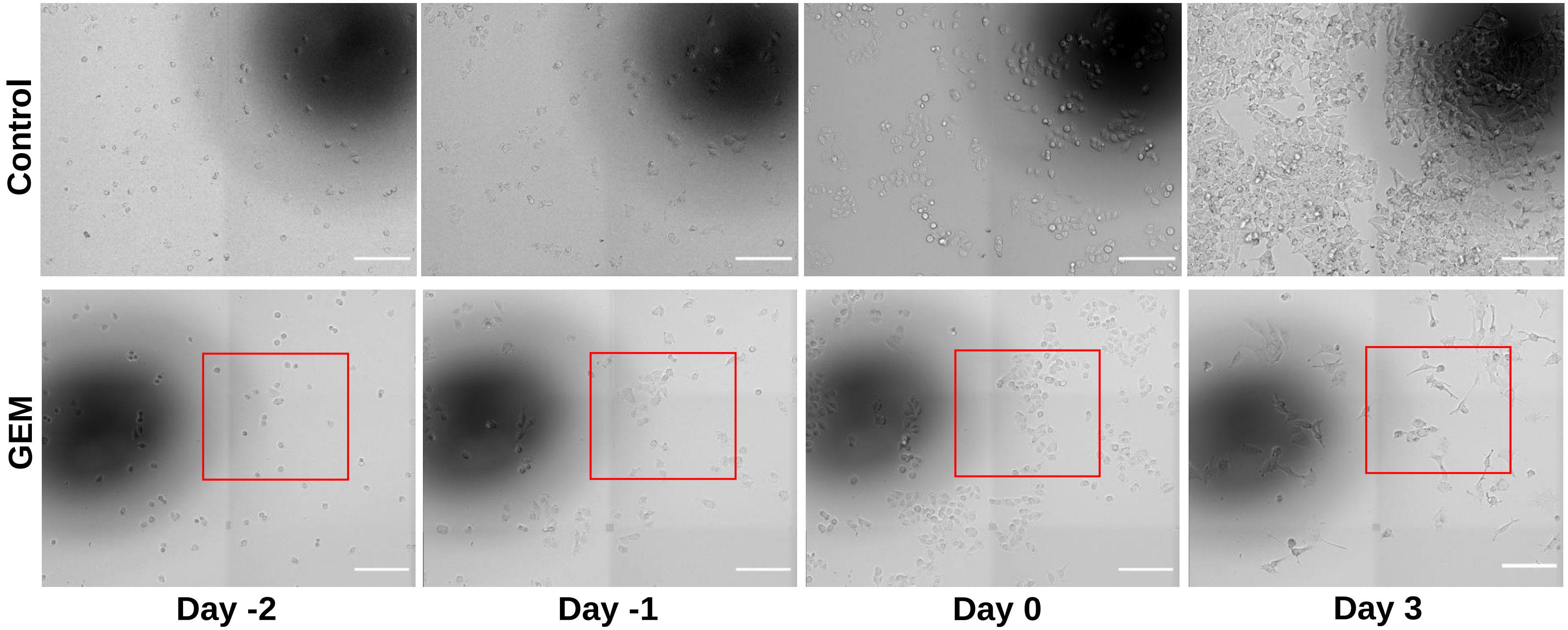

Supp. Figure S2: Monitoring melanoma cells with microscopy. A375 cells before (days -2 and -1), during (day 0) and after (day 3) GEM (10xIC_{50}) treatment were monitored with a microscope, which is capable of scanning the same area. The culture vessel was also marked with a black spot to further verify the selected location. Proliferating cells upon GEM treatment either died or entered a cell cycle arrest, leading to morphological changes (highlighted in red box). Scale bar: 100 µm.

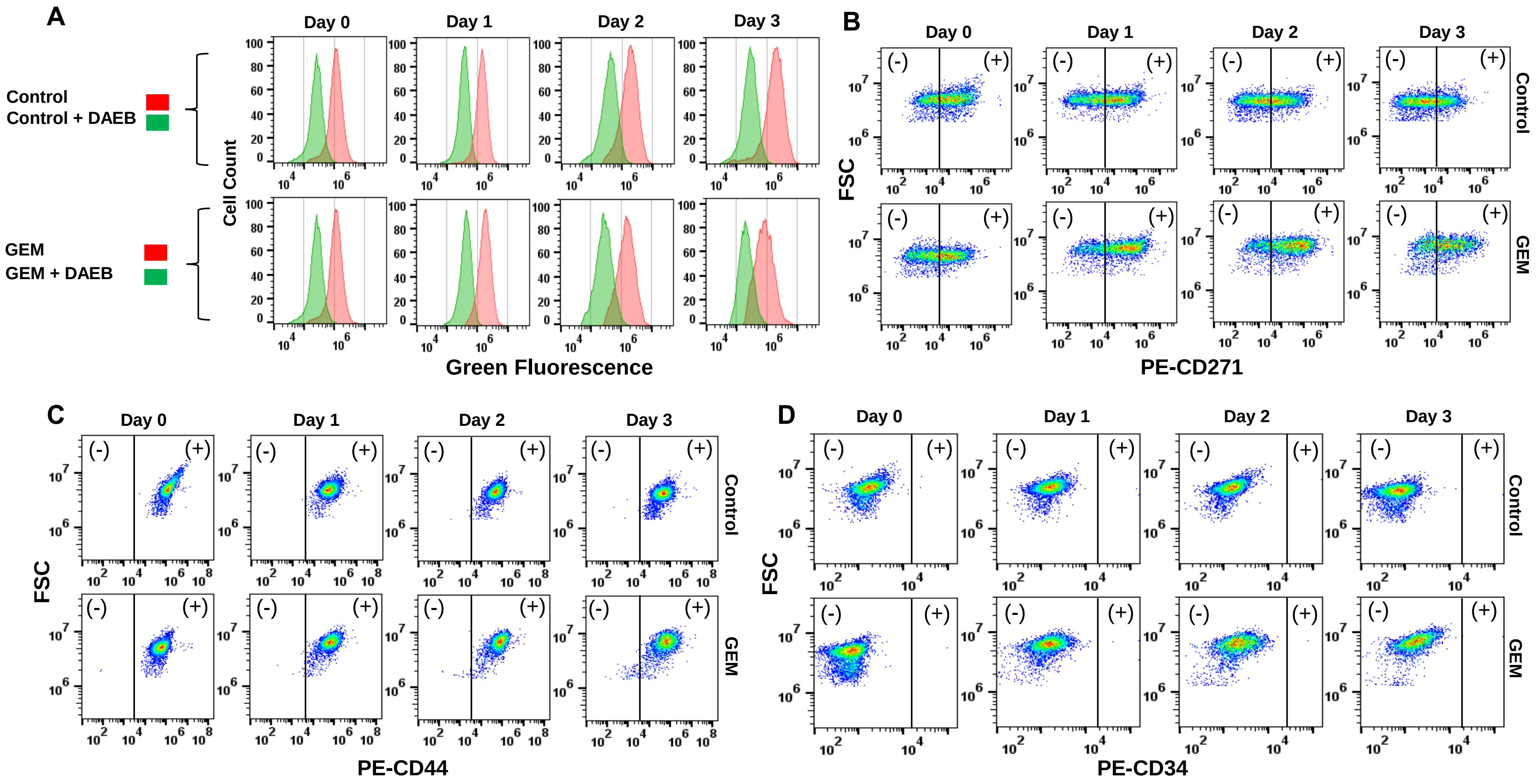

Supp. Figure S3: Stem cell biomarkers in A375 persister cells. (A) Melanoma cells were treated with GEM (10xIC_{50}) or left untreated for 3 days. Every day, the ALDH activity of the cells was assessed with the ALDEFLUOR assay and a flow cytometer. Cells treated with the ALDH inhibitor 4-(dimethylamino)benzaldehyde (DAEB) served as negative controls. **(B-D)** CD271, CD44 and CD34 biomarkers were detected with their respective conjugated antibodies. Cells treated with isotype controls of CD271, CD44 and CD34 were used to determine stem cell biomarker negative (-) and positive (+) populations. Live/dead staining was used to gate the live cells.

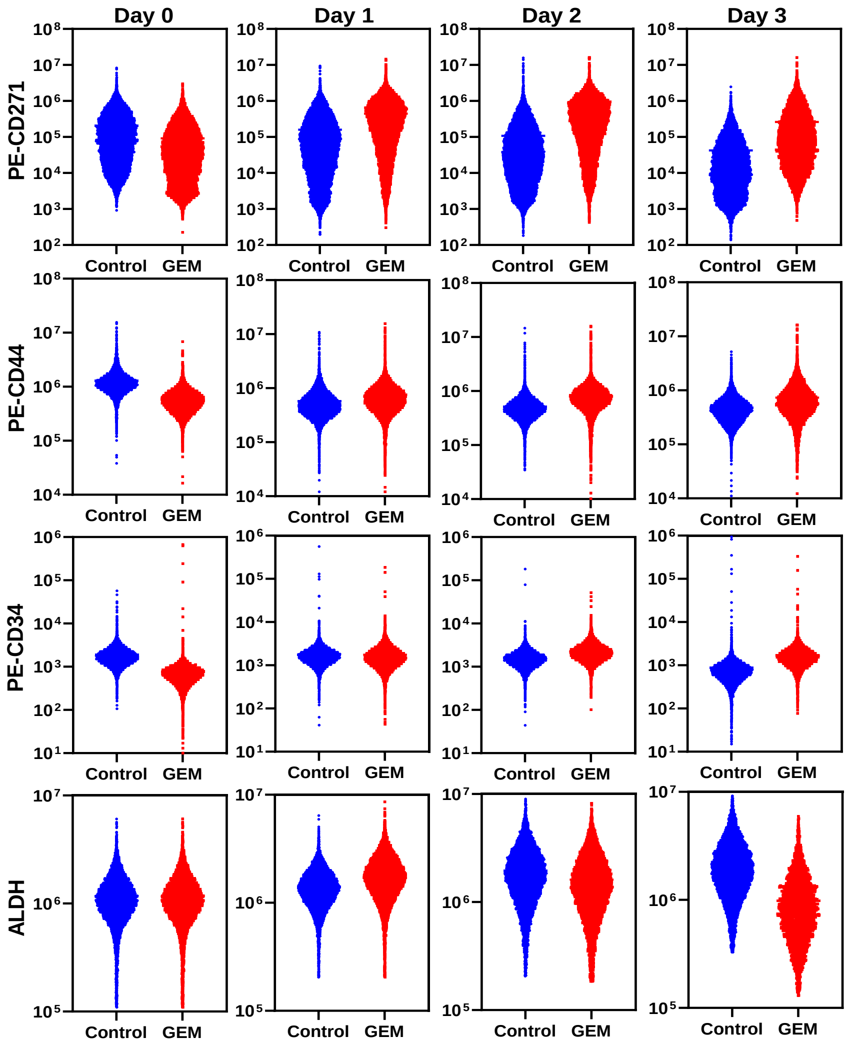

Supp. Figure S4: Stem cell biomarkers of persister and control groups. For better comparison, the flow cytometry data of persister and control groups from Figure S3 were plotted on the same graphs. Each point in the graph represents the fluorescence intensity of an individual cell.

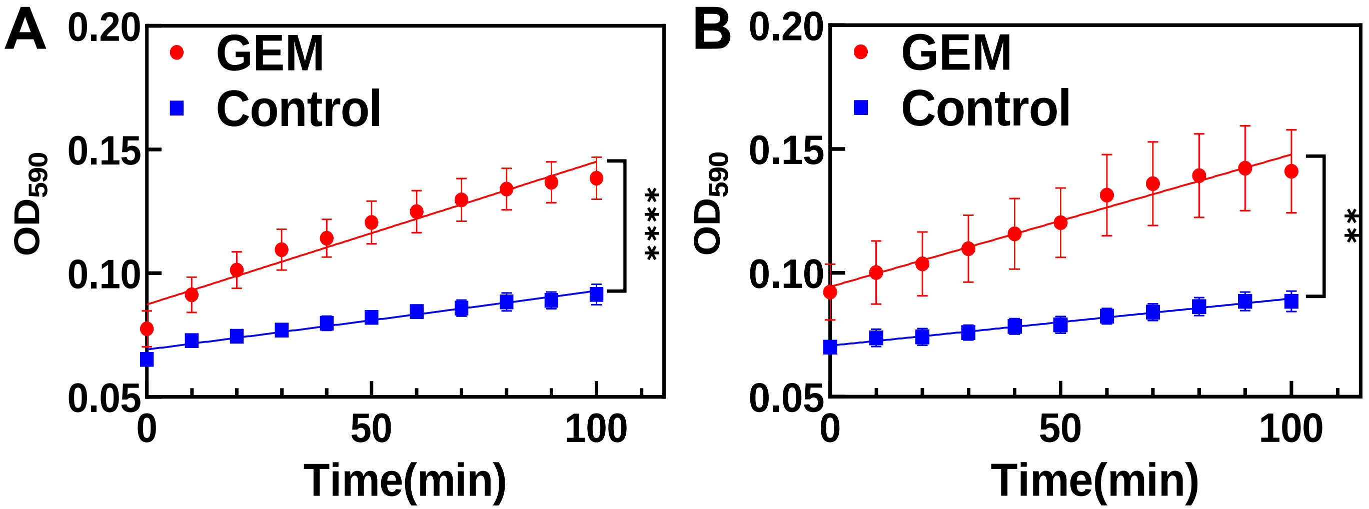

Supp. Figure S5. The basal ETC activities of GEM persister cells. (A) The regular or **(B)** modified MitoPlate assays were performed without adding any exogenous Krebs cycle substrates. Linear regression analysis was performed with F statistics using GraphPad Prism (**** P<0.0001, and ** P<0.01). N=4.

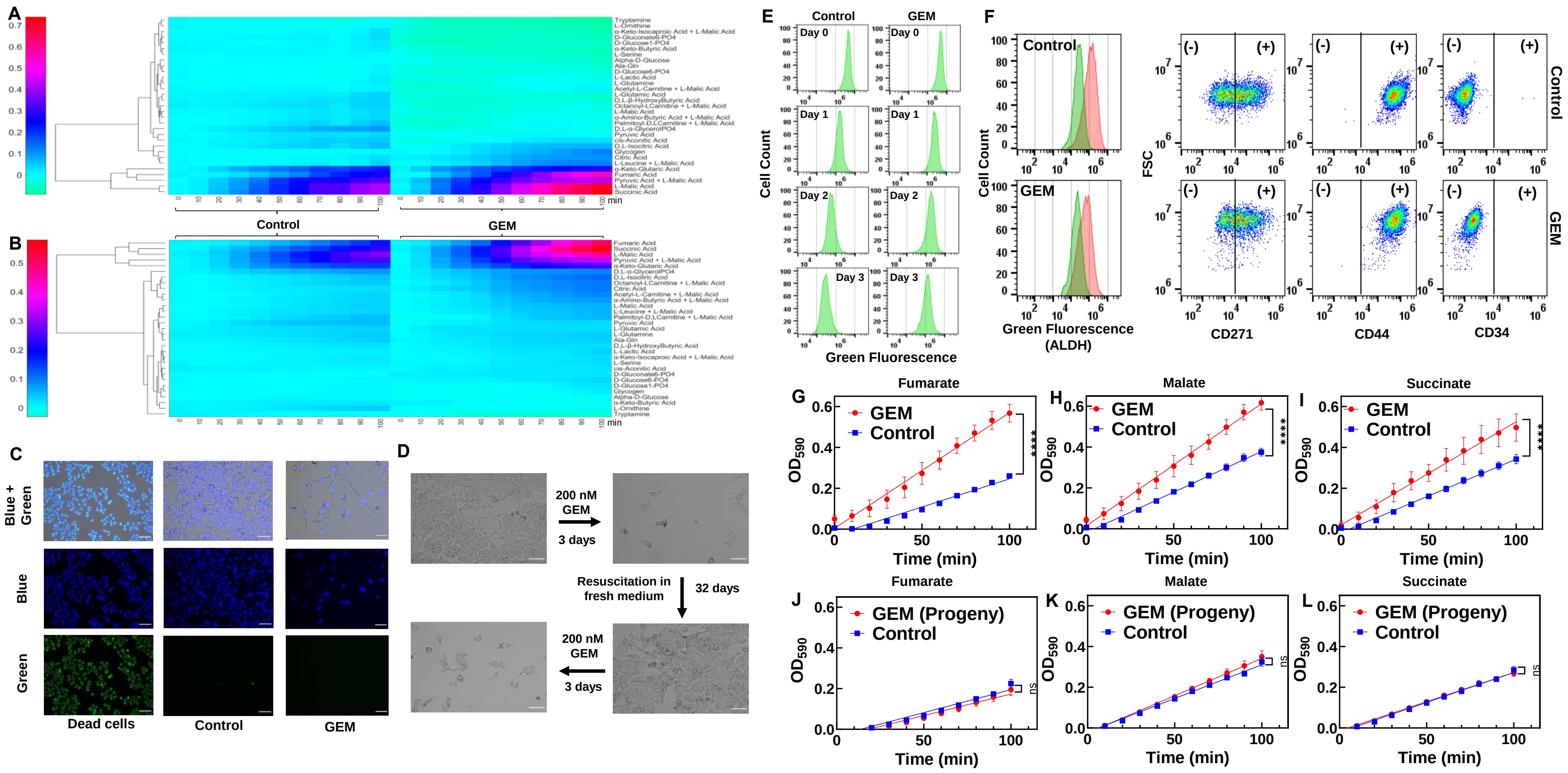

Supp. Figure S6: Effects of GEM concentration or treatment time on persister metabolism. (A-B) A375 cells were treated with GEM at 10 x IC_{50} concentration for 9 days **(A)** or at 100 x IC_{50} concentration for 3 days **(B)**, and then, 3 x 10^4 treated cells were transferred to each well of a phenotype microarray that also included a substrate and tetrazolium-based dye. For control, cells were treated with the solvent only. The consumption rates of the substrates were monitored with OD_{590} measurements at the indicated time points (for 100 min total). Unsupervised clustering of absorbance data was performed using MATLAB. N=4. **(C)** The cells surviving after GEM (100 x IC_{50}=200 nM) treatment were collected and transferred to fresh medium without GEM. The following day, cells were stained with ReadyProbes Cell Viability Imaging dyes to assess live (blue)

and dead (blue+green) cells. Dead cells were generated by treating the cells with 70% ethanol for 30 min. "Control" represents the live cells that did not receive GEM treatment. Scale bar: 100 µm. **(D)** Melanoma cells were treated with GEM (200 nM) for 3 days. After the treatment, cells were allowed to recover in fresh, drug-free growth medium and then treated with GEM (200 nM) again to demonstrate the sensitivity of the daughter cells to GEM. Scale bar: 100 µm. **(E)** Cells prestained with CFSE dye were treated with GEM (200 nM) or left untreated (control), and their fluorescence intensity was monitored at the indicated time points with flow cytometry. **(F)** Cells were treated with GEM (200 nM) or left untreated for 3 days. At the end of treatment, the stem cell biomarkers were assessed with the ALDEFLUOR assay and the conjugated antibodies. **(G-I)** The consumption rates for the selected substrates (4 mM) (fumarate, malate and succinate) of GEM (200 nM) persisters were measured with the modified MitoPlate assay. Linear regression analysis was performed with F statistics using GraphPad Prism (**** $P<0.0001$). N=4. **(J-L)** GEM (200 nM) persister cells were transferred to fresh medium without GEM to stimulate resuscitation. After the third passage, the daughter cells were collected, and their consumption rates for fumarate, malate and succinate were measured with the modified MitoPlate assay. Untreated cells were used as a control. N=4; ns: the slopes are not significantly different.

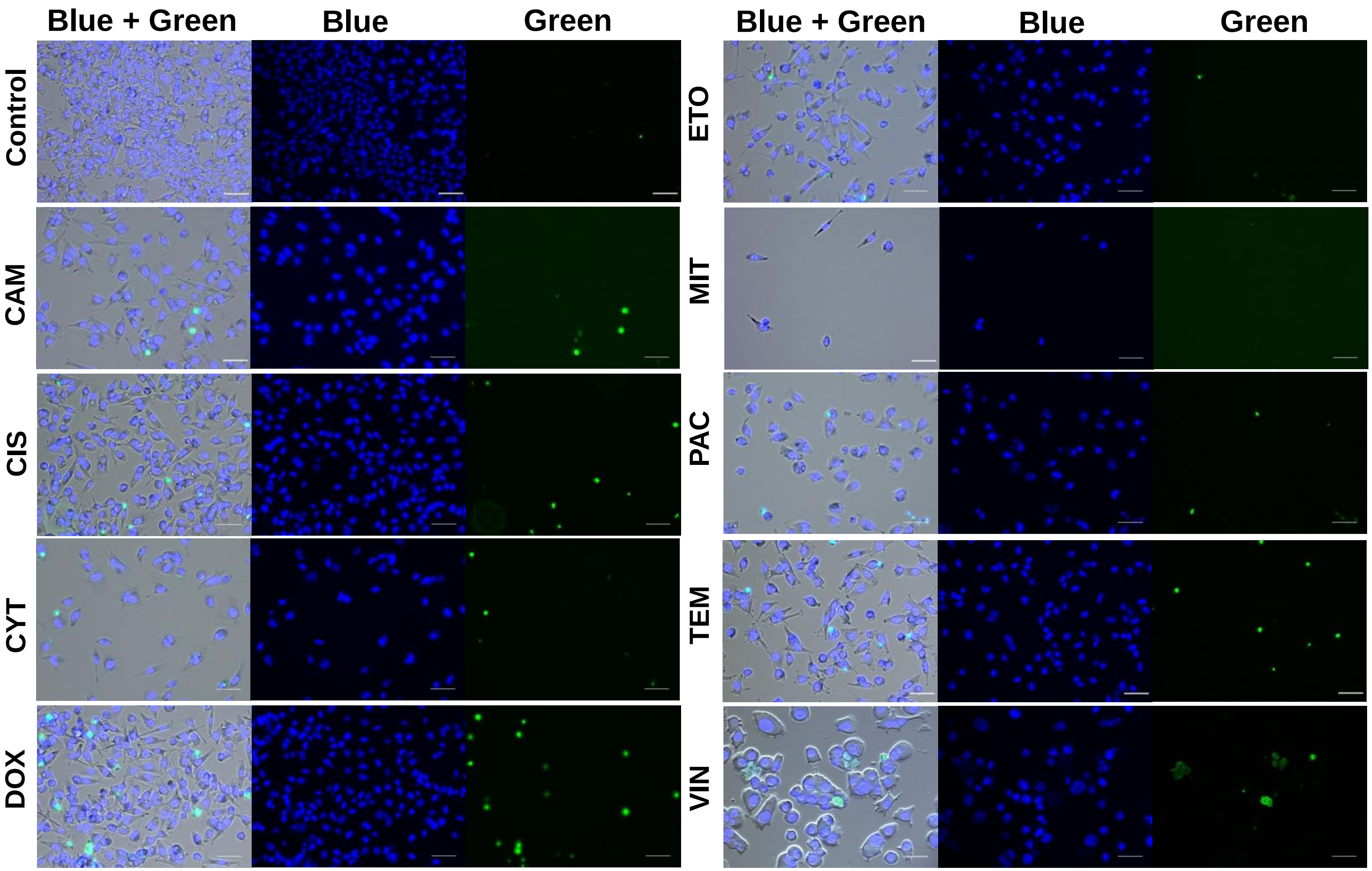

Supp. Figure S7: Assessing the viability of cells treated with various chemotherapeutic agents. A375 cells surviving the chemotherapeutic treatments were collected and transferred to fresh medium without drugs. The following day, the cells were stained with ReadyProbes Cell Viability Imaging dyes to assess live (blue) and dead (blue+green) cells. "Control" represents the live cells that did not receive GEM treatment. See Supp. Table S2 for the concentrations of chemotherapeutic agents. Scale bar: 100 µm.

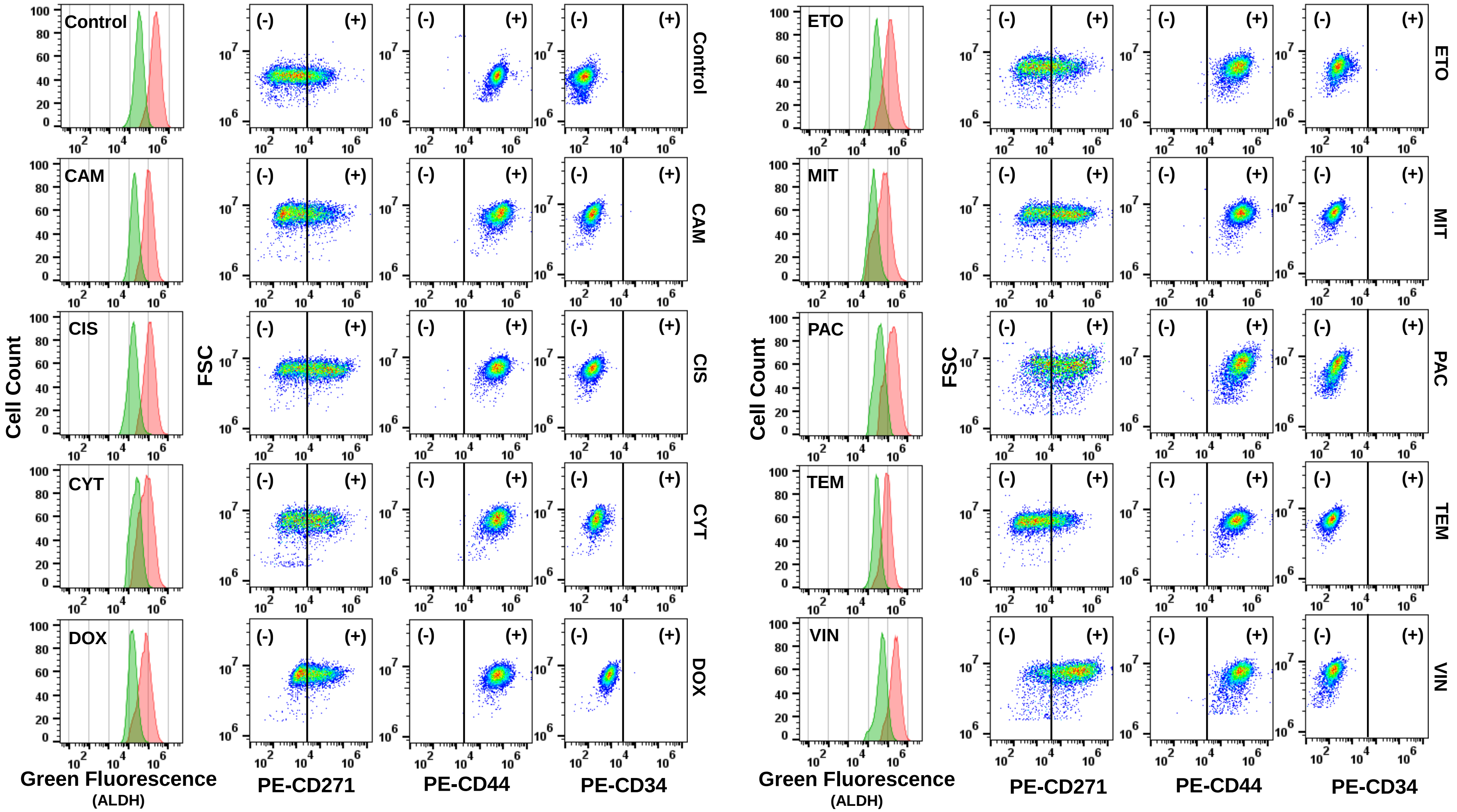

Supp. Figure S8: Assessing the stem cell biomarkers in A375 persister cells after chemotherapeutic treatments. A375 cells were treated with indicated chemotherapeutic agents or left untreated for 3 days. At the end of treatment, the ALDH activity of the cells was assessed with the ALDEFLUOR assay and a flow cytometer. Cells treated with the ALDH inhibitor (DAEB) served as negative controls (green). CD271, CD44 and CD34 biomarkers were detected with their respective conjugated antibodies. Cells treated with isotype controls of CD271, CD44 and CD34 were used to determine stem cell biomarker negative (-) and positive (+) populations. Live/dead staining was used to gate the live cells. See Supp. Table S2 for the concentrations of chemotherapeutic agents.

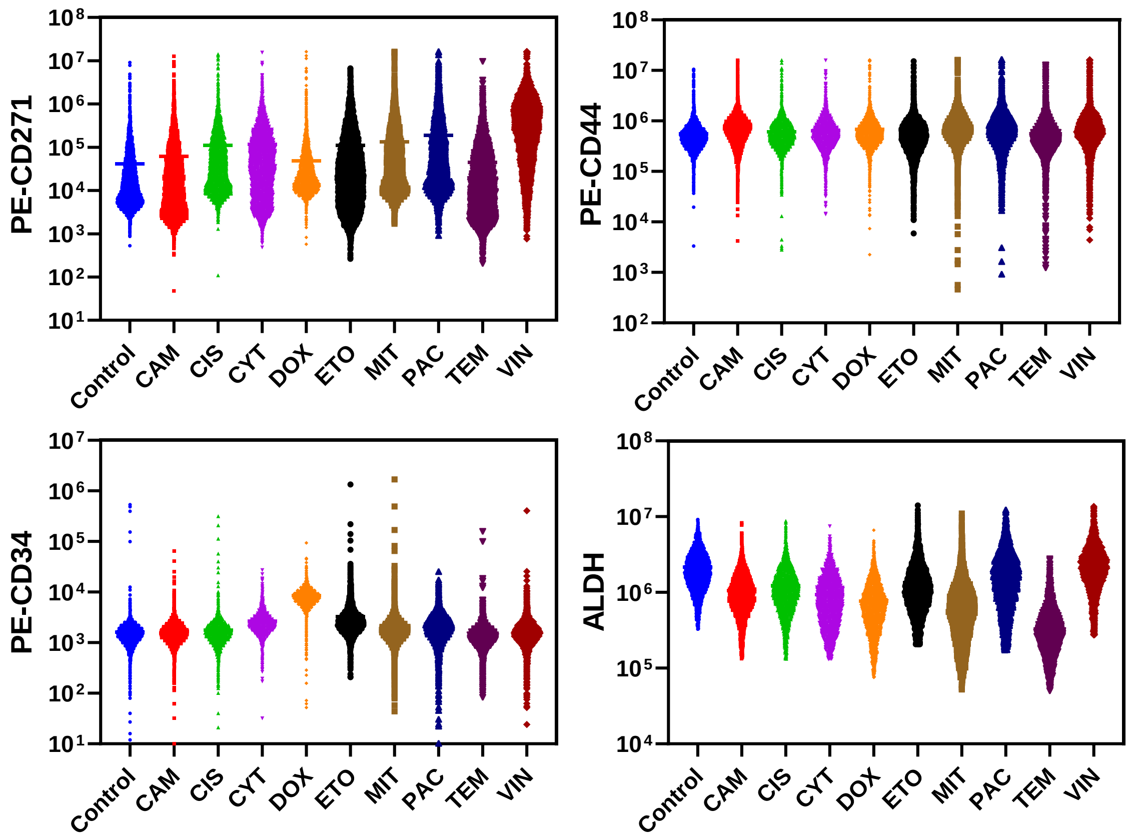

Supp. Figure S9: Stem cell biomarkers of persister and control groups. For better comparison, the flow cytometry data of persister and control groups from Figure S8 were plotted on the same graphs. Each point in the graph represents the fluorescence intensity of an individual cell.

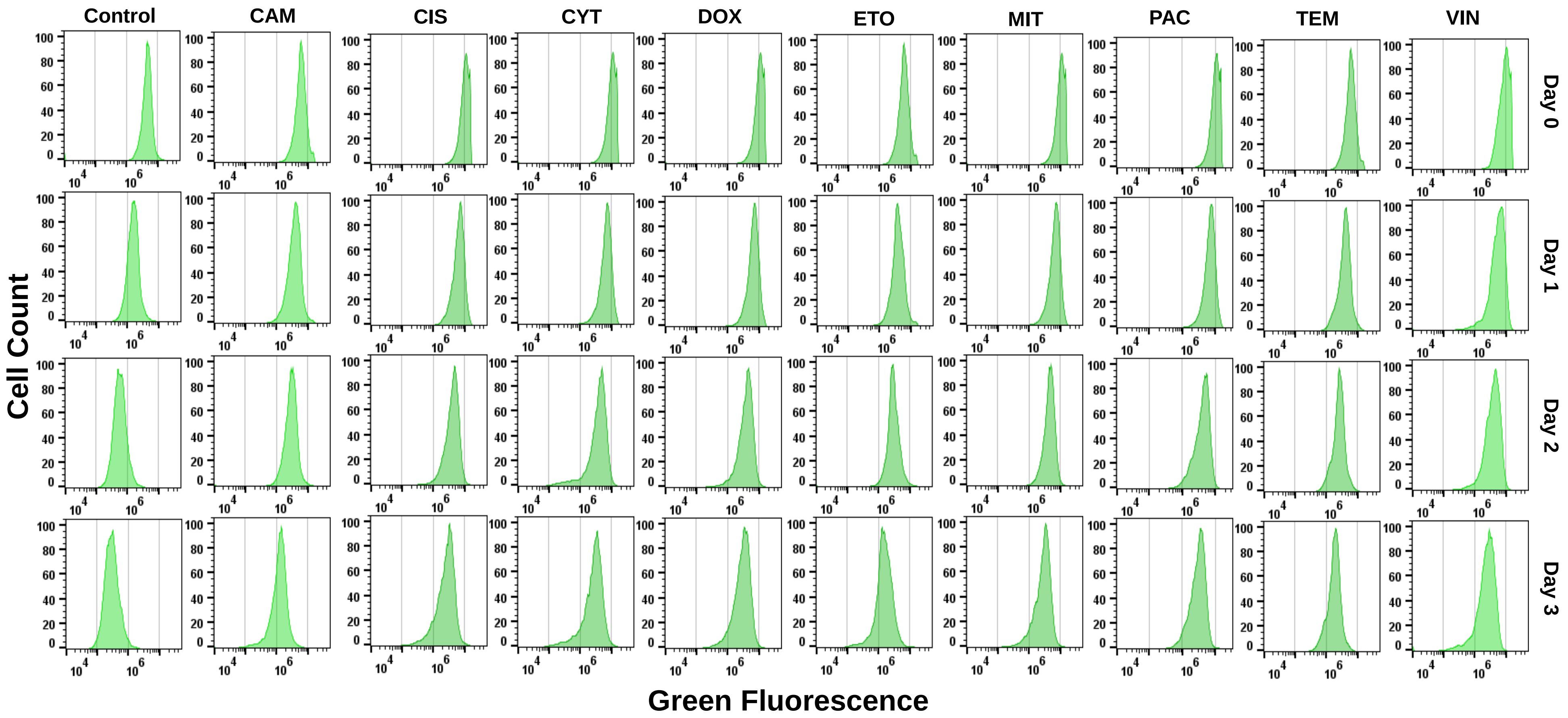

Supp. Figure S10: Assessing the growth of cells treated with chemotherapeutic agents. A375 cells prestained with CFSE dye were treated with indicated chemotherapeutic agents or left untreated (control), and their fluorescence intensity was monitored at the indicated time points with flow cytometry. See Supp. Table S2 for the concentrations of chemotherapeutic agents.

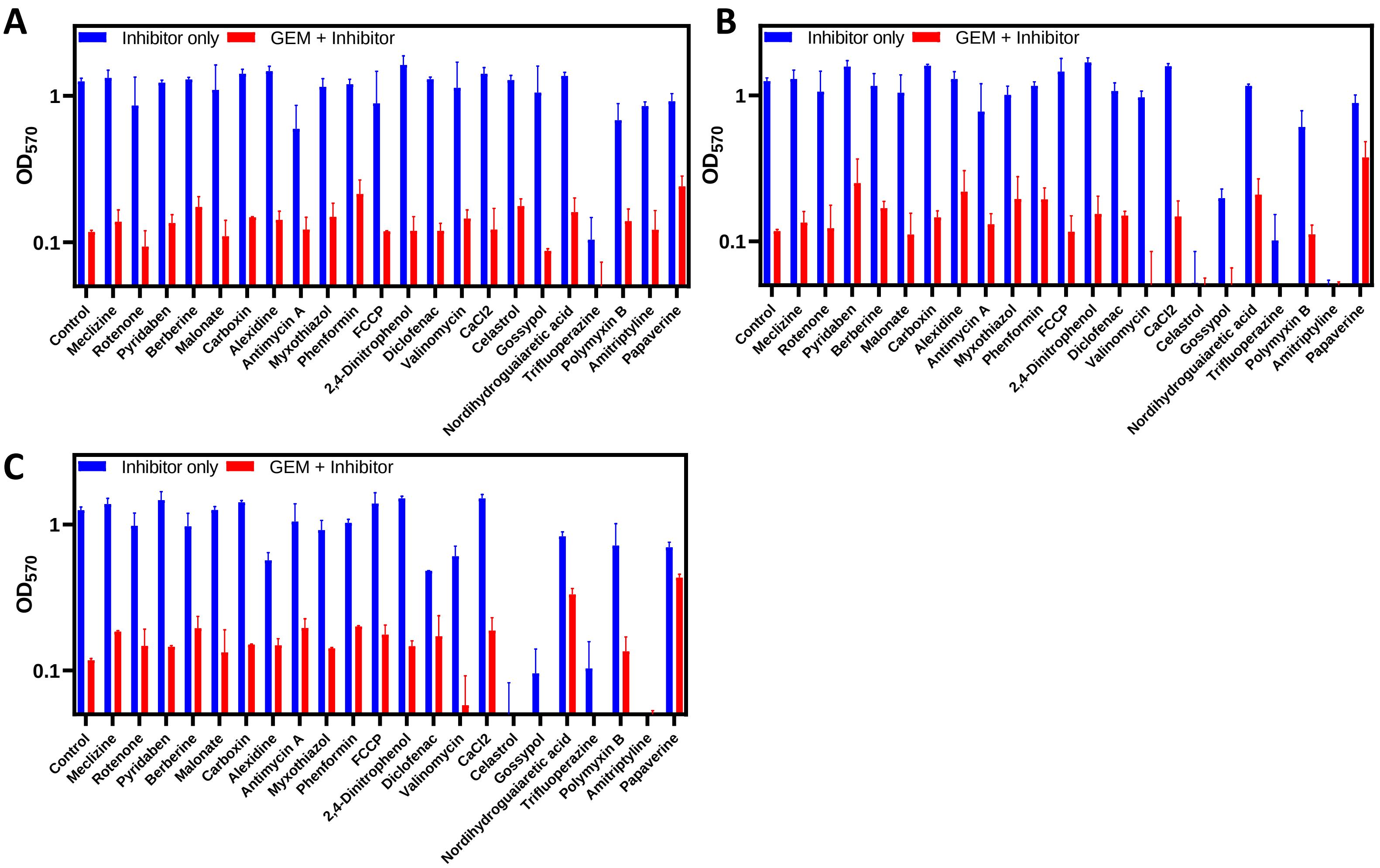

Supp. Figure S11: Screening for ETC inhibitors that affect persister survival. Melanoma cells were treated with various ETC (blue) or cotreated with GEM and ETC inhibitors (red) for 3 days. After the treatment, the media in the wells were replaced with fresh drug-free media. After 24 h of incubation, the MTT assay was conducted to assess cell viability by measuring the absorbance (OD_{570}) of all tested combinations with a plate reader. ETC inhibitors were obtained from I-1 plates (Biolog Inc., Hayward, CA). The graphs represent the cell viability assays conducted with various inhibitor concentrations (C_1, C_2 and C_3): **(A)** C_1, **(B)** C_2 and **(C)** C_3, where $C_1<C_2<C_3$. Note that this information was not disclosed by the company (Biolog, Inc.). N=2.

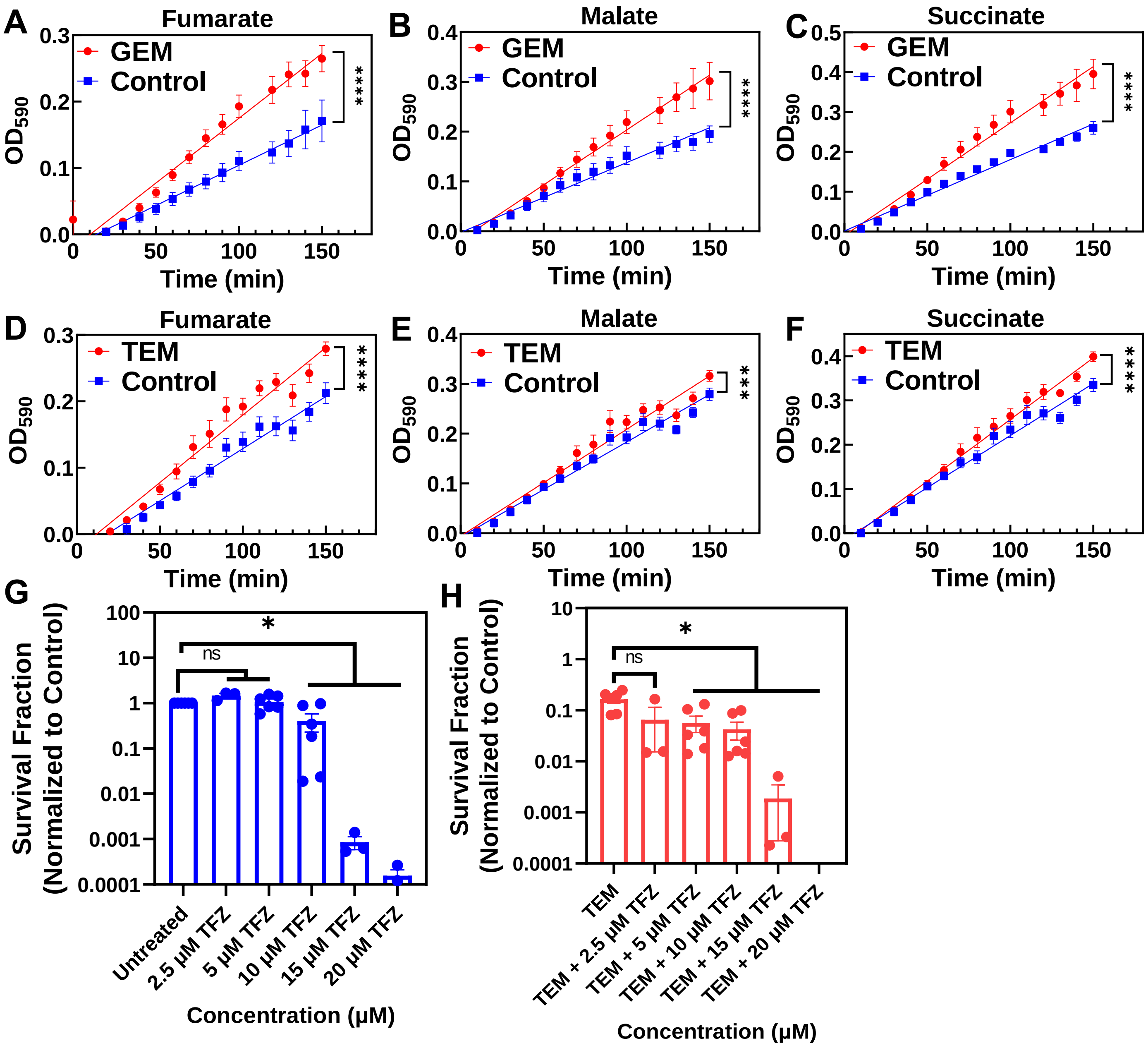

A
Fumarate
GEM
Control
OD590
Time (min)
****
B
Malate
GEM
Control
OD590
Time (min)
****
C
Succinate
GEM
Control
OD590
Time (min)
****
D
Fumarate
TEM
Control
OD590
Time (min)
****
E
Malate
TEM
Control
OD590
Time (min)
***
F
Succinate
TEM
Control
OD590
Time (min)
****
G
Survival Fraction (Normalized to Control)
*
ns
Untreated
2.5 μM TFZ
5 μM TFZ
10 μM TFZ
15 μM TFZ
20 μM TFZ
Concentration (μM)
H
Survival Fraction (Normalized to Control)
*
ns
TEM
TEM + 2.5 μM TFZ
TEM + 5 μM TFZ
TEM + 10 μM TFZ
TEM + 15 μM TFZ
TEM + 20 μM TFZ
Concentration (μM)

Supp. Figure S12: Increased consumption of TCA cycle substrates is observed in the RPMI-7951 cell line. **(A-F)** RPMI-7951 melanoma cells were treated with GEM (10xIC_{50}) or TEM (5xIC_{50}) (see Supp. Table S2) for 3 days. After the treatment, the consumption rates for the selected substrates (4 mM) (fumarate, malate and succinate) were measured with the modified MitoPlate assay. Linear regression analysis was performed using F statistics using GraphPad Prism (*** P<0.001, **** P<0.0001). **(G-H)** Cells were treated with TEM and/or TFZ (at indicated concentrations) for 3 days. After the treatments, the cells were collected and incubated in fresh, drug-free medium for 24 h, and then, the cell viability was assessed with STYO60 (red)/SYTOX (green) dyes using a flow cytometer. * represents a significant difference between the control and treatment groups (t-test, P<0.05). The total number of surviving cells were below the limit of detection for TFZ concentrations greater than 10 µM.

SUPPLEMENTARY TABLES

Supp. Table S1: Transcriptomics dataset analysis. Using the CMAP database, the 100 most downregulated genes in melanoma cells were selected using the z-score data matrix (see Materials and Methods). Genes directly or indirectly related to the cell cycle are highlighted with green color.

Gene	Gene Title
RPS4Y1	ribosomal protein S4, Y-linked 1
FABP5	fatty acid binding protein 5
GNAS	GNAS complex locus
KIF20A	kinesin family member 20A
S100A6	S100 calcium binding protein A6
KIAA1033	KIAA1033
NUSAP1	nucleolar and spindle associated protein 1
LSM5	LSM5 homolog, U6 small nuclear RNA and mRNA degradation associated
CDC20	cell division cycle 20
PCCB	propionyl-CoA carboxylase beta subunit
AKR7A2	aldo-keto reductase family 7, member A2
PCNA	proliferating cell nuclear antigen
ADI1	acireductone dioxygenase 1
VDAC1	voltage dependent anion channel 1
G3BP1	GTPase activating protein (SH3 domain) binding protein 1
CCNB2	cyclin B2
PRPF4	pre-mRNA processing factor 4
KIF14	kinesin family member 14
IARS2	isoleucyl-tRNA synthetase 2, mitochondrial
PSIP1	PC4 and SFRS1 interacting protein 1
TXNDC9	thioredoxin domain containing 9
C2CD5	C2 calcium-dependent domain containing 5
XPO7	exportin 7
NRIP1	nuclear receptor interacting protein 1
TIMM9	translocase of inner mitochondrial membrane 9 homolog (yeast)
ADH5	alcohol dehydrogenase 5 (class III), chi polypeptide
HMG20B	high mobility group 20B
HEATR1	HEAT repeat containing 1
IFRD2	interferon-related developmental regulator 2
OXA1L	oxidase (cytochrome c) assembly 1-like
NR2F6	nuclear receptor subfamily 2 group F member 6
CCDC86	coiled-coil domain containing 86
CD58	CD58 molecule
STUB1	STIP1 homology and U-box containing protein 1, E3 ubiquitin protein ligase
PGAM1	phosphoglycerate mutase 1
BAG3	BCL2 associated athanogene 3
CCNB1	cyclin B1
TLE1	transducin like enhancer of split 1
CDC25B	cell division cycle 25B
ENOPH1	enolase-phosphatase 1
PWP1	PWP1 homolog, endonuclein
CD320	CD320 molecule
RBM34	RNA binding motif protein 34
DYNLT3	dynein, light chain, Tctex-type 3

MACF1	microtubule-actin crosslinking factor 1
EPHA3	EPH receptor A3
MAP4K4	mitogen-activated protein kinase kinase kinase kinase 4
ITGB1BP1	integrin subunit beta 1 binding protein 1
MRPS16	mitochondrial ribosomal protein S16
DLD	dihydrolipoamide dehydrogenase
TOP2A	topoisomerase (DNA) II alpha
TSPAN4	tetraspanin 4
PIN1	peptidylprolyl cis/trans isomerase, NIMA-interacting 1
STMN1	stathmin 1
LYPLA1	lysophospholipase I
UBE2C	ubiquitin conjugating enzyme E2C
RAD51C	RAD51 paralog C
MELK	maternal embryonic leucine zipper kinase
RPIA	ribose 5-phosphate isomerase A
CIAPIN1	cytokine induced apoptosis inhibitor 1
VAT1	vesicle amine transport 1
HAT1	histone acetyltransferase 1
HSD17B11	hydroxysteroid (17-beta) dehydrogenase 11
RFX5	regulatory factor X5
TRAPPC3	trafficking protein particle complex 3
SLC25A46	solute carrier family 25 member 46
CCNA2	cyclin A2
CDK1	cyclin-dependent kinase 1
ATG3	autophagy related 3
MCM3	minichromosome maintenance complex component 3
IKBKAP	inhibitor of kappa light polypeptide gene enhancer in B-cells, kinase complex-associated protein
HSPA8	heat shock protein family A (Hsp70) member 8
CSRP1	cysteine and glycine rich protein 1
FIS1	fission, mitochondrial 1
POLG2	polymerase (DNA directed), gamma 2, accessory subunit
YKT6	YKT6 v-SNARE homolog (S. cerevisiae)
HLA-DRA	major histocompatibility complex, class II, DR alpha
CDC42	cell division cycle 42
BNIP3	BCL2/adenovirus E1B 19kDa interacting protein 3
CYCS	cytochrome c, somatic
CNDP2	CNDP dipeptidase 2 (metallopeptidase M20 family)
DNM1L	dynamin 1-like
MTERF3	mitochondrial transcription termination factor 3
LBR	lamin B receptor
SLC35F2	solute carrier family 35 member F2
HMGA2	high mobility group AT-hook 2
CANT1	calcium activated nucleotidase 1
ITGAE	integrin subunit alpha E
DNAJA3	DnaJ heat shock protein family (Hsp40) member A3
RPA3	replication protein A3
CCNF	cyclin F
ITGB5	integrin subunit beta 5
CREB1	cAMP responsive element binding protein 1

CHIC2	cysteine rich hydrophobic domain 2
PARP2	poly(ADP-ribose) polymerase 2
MLLT11	myeloid/lymphoid or mixed-lineage leukemia; translocated to, 11
PPP2R3C	protein phosphatase 2 regulatory subunit B", gamma
CREG1	cellular repressor of E1A stimulated genes 1
USP7	ubiquitin specific peptidase 7 (herpes virus-associated)
ACOT9	acyl-CoA thioesterase 9

Supp. Table S2: Concentrations of chemotherapeutic agents. The table represents the IC_{50} and treatment concentrations for the indicated chemotherapeutic agents used in this study. The IC_{50} were obtained from "The Genomics of Drug Sensitivity in Cancer Project" website.*

Chemotherapeutic agents	Classification	IC50 (µM)	Working concentration (µM)
Campothecin	Alkaloid	0.01	0.1
Cisplatin	Alkylating agent	13	130
Cytarabine	Antimetabolite	0.5	5
Doxorubicin	Anthracycline antibiotic	0.024	0.24
Etoposide	Alkaloid	6	60
Gemcitabine	Antimetabolite	0.002	0.02
Mitomycin-C	Alkylating agent	0.26	2.6
Paclitaxel	Antimicrotubule agent	0.0147	0.147
Temozolomide	Alkylating agent	272	1360
Vinorelbine	Alkaloid	0.27	2.7

* Yang, W. *et al.* Genomics of Drug Sensitivity in Cancer (GDSC): A resource for therapeutic biomarker discovery in cancer cells. *Nucleic Acids Res.* **41**, (2013).

Supp. Table S3: Metabolomics dataset for GEM treated A375 cells. GEM-treated and untreated cells were collected for MS analysis to measure their metabolite contents. The fold change for each metabolite was calculated by normalizing the GEM data to the control data.

Super Pathway	Sub Pathway	Biochemical Name	Fold Change	p-value	q-value
Amino Acid	Glycine, Serine and Threonine Metabolism	glycine	0.53	1.720E-06	0.000
		sarcosine	0.11	1.129E-06	0.000
		dimethylglycine	0.59	1.000E-04	0.000
		betaine	0.57	1.157E-05	0.000
		serine	0.67	6.000E-04	0.001
		N-acetylserine	0.34	2.320E-08	0.000
		threonine	1.58	4.000E-04	0.001
		N-acetylthreonine	0.53	3.000E-04	0.001
	Alanine and Aspartate Metabolism	alanine	1.11	1.953E-01	0.136
		N-acetylalanine	0.65	6.000E-04	0.001
		aspartate	1.06	5.664E-01	0.309
		N-acetylaspartate (NAA)	0.80	7.800E-03	0.010
		asparagine	1.19	5.440E-02	0.048
		N-acetylasparagine	0.79	8.640E-02	0.070
		hydroxyasparagine**	0.47	2.400E-03	0.004
	Glutamate Metabolism	glutamate	0.94	4.031E-01	0.241
		glutamine	0.99	9.233E-01	0.433
		alpha-ketoglutaramate*	0.63	7.370E-02	0.062
		N-acetylglutamate	0.48	2.420E-07	0.000
		N-acetylglutamine	0.75	6.000E-03	0.008
		4-hydroxyglutamate	0.30	1.118E-10	0.000
		glutamate, gamma-methyl ester	0.91	7.535E-01	0.381
		pyroglutamine*	0.34	6.481E-08	0.000
		N-acetyl-aspartyl-glutamate (NAAG)	0.52	2.344E-05	0.000
		beta-citrylglutamate	0.64	4.207E-05	0.000
		carboxyethyl-GABA	6.24	1.588E-10	0.000
		S-1-pyrroline-5-carboxylate	0.77	5.481E-01	0.302
	Histidine Metabolism	histidine	2.04	6.383E-06	0.000
		1-methylhistidine	2.46	1.000E-04	0.000
		3-methylhistidine	2.08	9.000E-04	0.002
		N-acetylhistidine	2.06	2.359E-06	0.000
		N-acetyl-3-methylhistidine*	1.86	1.000E-04	0.000
		N-acetyl-1-methylhistidine*	1.96	3.200E-03	0.005
		trans-urocanate	0.88	4.313E-01	0.253
		imidazole propionate	0.18	9.405E-11	0.000
		formiminoglutamate	0.83	2.202E-01	0.150
		imidazole lactate	0.14	4.368E-11	0.000
		carnosine	1.90	1.186E-05	0.000
		1-methyl-4-imidazoleacetate	0.51	2.191E-05	0.000

		1-methyl-5-imidazoleacetate	**0.15**	8.930E-11	0.000
		1-ribosyl-imidazoleacetate*	**0.27**	2.649E-06	0.000
		4-imidazoleacetate	1.02	7.578E-01	0.381
		histidine methyl ester	1.11	2.492E-01	0.166
	Lysine Metabolism	lysine	0.98	8.579E-01	0.417
		N2-acetyllysine	**3.99**	1.291E-05	0.000
		N6-acetyllysine	**0.66**	5.000E-04	0.001
		N6-methyllysine	**1.26**	3.070E-02	0.031
		N6,N6-dimethyllysine	**0.72**	5.340E-02	0.047
		N6,N6,N6-trimethyllysine	**1.63**	3.749E-05	0.000
		hydroxy-N6,N6,N6-trimethyllysine*	**1.45**	4.500E-03	0.006
		5-hydroxylysine	**1.59**	1.550E-02	0.017
		5-(galactosylhydroxy)-L-lysine	**2.16**	1.082E-05	0.000
		fructosyllysine	1.17	3.992E-01	0.239
		saccharopine	**3.57**	5.785E-05	0.000
		2-aminoadipate	**1.90**	7.300E-03	0.009
		glutarylcarnitine (C5-DC)	**1.80**	5.120E-02	0.046
		pipecolate	0.93	5.261E-01	0.295
		6-oxopiperidine-2-carboxylate	**0.82**	6.230E-02	0.054
		cadaverine	**0.87**	6.200E-03	0.008
		N-acetyl-cadaverine	0.44	1.661E-01	0.119
		5-aminovalerate	**0.69**	1.000E-03	0.002
		N,N,N-trimethyl-5-aminovalerate	**0.44**	1.223E-06	0.000
	Phenylalanine Metabolism	phenylalanine	1.09	2.020E-01	0.139
		N-acetylphenylalanine	**1.24**	7.020E-02	0.059
		1-carboxyethylphenylalanine	**0.77**	5.130E-02	0.046
		phenylpyruvate	1.35	3.038E-01	0.194
		phenyllactate (PLA)	**0.74**	8.940E-02	0.072
		phenethylamine	1.60	1.651E-01	0.119
	Tyrosine Metabolism	tyrosine	1.18	1.045E-01	0.082
		N-acetyltyrosine	**1.65**	3.150E-05	0.000
		1-carboxyethyltyrosine	**0.41**	4.248E-06	0.000
		4-hydroxyphenylpyruvate	**1.28**	4.290E-02	0.040
		3-(4-hydroxyphenyl)lactate	**0.62**	2.818E-05	0.000
		phenol sulfate	**1.45**	4.350E-02	0.041
		3-methoxytyrosine	**2.35**	1.000E-04	0.000
		o-Tyrosine	**6.27**	6.572E-05	0.000
		O-methyltyrosine	**0.55**	9.599E-06	0.000
	Tryptophan Metabolism	tryptophan	**1.31**	6.800E-03	0.009
		N-acetyltryptophan	1.20	1.551E-01	0.114
		C-glycosyltryptophan	**0.71**	1.180E-02	0.014
		tryptophan betaine	**0.39**	1.818E-05	0.000
		kynurenine	**2.02**	5.896E-06	0.000

		kynurenate	1.96	6.500E-03	0.008
		serotonin	2.65	5.000E-04	0.001
		tryptamine	1.21	4.761E-01	0.274
		indolelactate	1.51	7.460E-02	0.062
		indoleacetate	1.27	2.042E-01	0.140
	Leucine, Isoleucine and Valine Metabolism	leucine	1.13	8.110E-02	0.066
		N-acetylleucine	1.29	9.660E-02	0.076
		1-carboxyethylleucine	1.16	1.995E-01	0.138
		4-methyl-2-oxopentanoate	0.39	3.735E-05	0.000
		alpha-hydroxyisocaproate	0.85	2.544E-01	0.168
		isovalerylglycine	0.76	3.955E-01	0.238
		isovalerylcarnitine (C5)	0.62	2.510E-02	0.026
		beta-hydroxyisovalerate	0.58	4.000E-04	0.001
		beta-hydroxyisovaleroylcarnitine	0.72	2.700E-03	0.004
		3-methylglutaconate	0.78	1.180E-02	0.014
		isoleucine	1.08	2.493E-01	0.166
		N-acetylisoleucine	0.89	8.353E-01	0.409
		1-carboxyethylisoleucine	0.63	1.500E-03	0.003
		3-methyl-2-oxovalerate	0.41	6.100E-03	0.008
		2-hydroxy-3-methylvalerate	0.89	4.476E-01	0.260
		2-methylbutyrylcarnitine (C5)	0.52	3.000E-04	0.001
		2-methylbutyrylglycine	0.68	8.120E-02	0.066
		tiglylcarnitine (C5:1-DC)	0.68	3.200E-03	0.005
		3-hydroxy-2-ethylpropionate	0.91	8.408E-01	0.411
		ethylmalonate	0.35	2.953E-07	0.000
		methylsuccinate	0.63	3.400E-03	0.005
		valine	1.11	1.276E-01	0.097
		N-acetylvaline	1.08	4.188E-01	0.247
		1-carboxyethylvaline	0.55	1.600E-03	0.003
		3-methyl-2-oxobutyrate	0.39	1.900E-03	0.003
		alpha-hydroxyisovalerate	0.96	6.967E-01	0.356
		isobutyrylcarnitine (C4)	0.42	1.640E-05	0.000
		3-hydroxyisobutyrate	1.08	6.664E-01	0.348
		2,3-dihydroxy-2-methylbutyrate	0.32	6.113E-07	0.000
	Methionine, Cysteine, SAM and Taurine Metabolism	methionine	1.15	6.960E-02	0.059
		N-acetylmethionine	1.71	7.000E-04	0.001
		N-formylmethionine	0.93	4.241E-01	0.249
		S-methylmethionine	1.44	2.529E-01	0.168
		methionine sulfone	1.46	1.450E-02	0.016
		methionine sulfoxide	3.87	3.464E-07	0.000
		N-acetylmethionine sulfoxide	3.21	7.478E-05	0.000
		S-adenosylmethionine (SAM)	1.18	6.120E-02	0.053
		S-adenosylhomocysteine (SAH)	0.68	7.261E-05	0.000

		2,3-dihydroxy-5-methylthio-4-pentenoate (DMTPA)*	**0.77**	2.030E-02	0.021
		homocysteine	**0.65**	4.530E-02	0.042
		cystathionine	**0.19**	4.333E-11	0.000
		cysteine	**1.38**	3.720E-02	0.036
		N-acetylcysteine	0.87	4.612E-01	0.267
		S-methylcysteine sulfoxide	1.23	3.129E-01	0.198
		S-carboxyethylcysteine	**3.79**	3.215E-08	0.000
		hypotaurine	**0.58**	2.000E-03	0.003
		taurine	**0.52**	1.400E-03	0.002
		N-acetyltaurine	**0.37**	7.960E-07	0.000
	Urea cycle; Arginine and Proline Metabolism	arginine	**1.55**	9.000E-04	0.002
		argininosuccinate	0.77	2.096E-01	0.144
		ornithine	**4.81**	1.035E-05	0.000
		3-amino-2-piperidone	**6.24**	6.616E-05	0.000
		2-oxoarginine*	1.20	2.017E-01	0.139
		citrulline	**1.15**	9.590E-02	0.076
		proline	**0.52**	2.000E-04	0.000
		dimethylarginine (SDMA + ADMA)	**1.49**	6.300E-03	0.008
		N-acetylarginine	**1.17**	3.570E-02	0.035
		N-delta-acetylornithine	**1.54**	1.300E-03	0.002
		trans-4-hydroxyproline	**1.80**	1.945E-05	0.000
		pro-hydroxy-pro	**0.56**	1.760E-02	0.019
		N-methylproline	**0.53**	1.100E-03	0.002
		N,N,N-trimethyl-alanylproline betaine (TMAP)	**1.44**	5.700E-03	0.008
		N-monomethylarginine	**1.45**	4.080E-02	0.039
	Creatine Metabolism	creatine	**0.72**	2.000E-04	0.001
		creatinine	**0.75**	6.000E-03	0.008
		creatine phosphate	**0.61**	7.000E-04	0.001
	Polyamine Metabolism	putrescine	**0.28**	5.825E-07	0.000
		N-acetylputrescine	0.65	3.683E-01	0.224
		N-acetyl-isoputreanine	**2.34**	6.216E-07	0.000
		spermidine	**1.84**	7.499E-05	0.000
		N('1)-acetylspermidine	**6.76**	4.302E-08	0.000
		diacetylspermidine*	**1.69**	6.000E-04	0.001
		spermine	**0.53**	5.020E-02	0.046
		N(1)-acetylspermine	**3.04**	1.081E-06	0.000
		N1,N12-diacetylspermine	**3.34**	9.971E-06	0.000
		5-methylthioadenosine (MTA)	1.06	3.538E-01	0.217
		4-acetamidobutanoate	**0.79**	8.060E-02	0.066
	Guanidino and Acetamido Metabolism	1-methylguanidine	**1.46**	7.520E-02	0.063
		4-guanidinobutanoate	**0.37**	1.990E-10	0.000
	Glutathione Metabolism	glutathione, reduced (GSH)	**1.22**	5.110E-02	0.046

		glutathione, oxidized (GSSG)	1.04	6.088E-01	0.326
		cyclic dGSH	1.10	2.839E-01	0.184
		cysteine-glutathione disulfide	1.28	2.298E-01	0.155
		S-methylglutathione	**1.33**	3.700E-02	0.036
		cysteinylglycine	**1.36**	1.400E-03	0.002
		5-oxoproline	**1.16**	5.890E-02	0.052
		2-hydroxybutyrate/2-hydroxyisobutyrate	0.92	2.288E-01	0.155
		ophthalmate	**9.26**	1.349E-07	0.000
		S-(1,2-dicarboxyethyl)glutathione	**0.75**	1.810E-02	0.019
		4-hydroxy-nonenal-glutathione	0.85	3.196E-01	0.202
		CoA-glutathione*	1.07	5.684E-01	0.309
Peptide	Gamma-glutamyl Amino Acid	gamma-glutamylcysteine	1.13	3.075E-01	0.196
		gamma-glutamylglutamate	0.85	1.564E-01	0.114
		gamma-glutamylglutamine	**2.84**	2.232E-06	0.000
		gamma-glutamylhistidine	**3.63**	1.000E-04	0.000
		gamma-glutamylisoleucine*	**2.45**	1.343E-07	0.000
		gamma-glutamylleucine	**3.71**	2.141E-07	0.000
		gamma-glutamylmethionine	**1.57**	1.250E-02	0.014
		gamma-glutamylphenylalanine	**1.76**	2.000E-04	0.000
		gamma-glutamylthreonine	**8.52**	1.104E-08	0.000
		gamma-glutamyltryptophan	**1.19**	6.540E-02	0.056
		gamma-glutamyltyrosine	**1.64**	8.500E-03	0.010
		gamma-glutamylvaline	**3.32**	5.218E-08	0.000
	Dipeptide	alanylleucine	**2.16**	1.000E-03	0.002
		glycylisoleucine	**3.83**	4.799E-05	0.000
		glycylleucine	**2.37**	1.100E-03	0.002
		glycylvaline	**2.03**	4.700E-03	0.006
		isoleucylglycine	**4.22**	4.277E-05	0.000
		leucylglycine	**3.53**	4.000E-04	0.001
		phenylalanylalanine	**3.11**	4.000E-04	0.001
		phenylalanylglycine	**2.81**	2.000E-04	0.000
		prolylglycine	**1.30**	3.440E-02	0.034
		threonylphenylalanine	**1.66**	1.840E-02	0.020
		tryptophylglycine	**3.28**	8.662E-05	0.000
		tyrosylglycine	**3.14**	4.000E-04	0.001
		valylglutamine	**1.77**	2.800E-03	0.004
		valylglycine	**4.24**	2.000E-04	0.000
		valylleucine	**4.40**	1.000E-04	0.000
		leucylglutamine*	**3.66**	3.415E-05	0.000
	Acetylated Peptides	phenylacetylglycine	**0.61**	1.830E-02	0.020
Carbohydrate	Glycolysis, Gluconeogenesis, and Pyruvate Metabolism	glucose	1.42	6.397E-01	0.339
		glucose 6-phosphate	1.61	1.063E-01	0.082

		fructose 1,6-diphosphate/glucose 1,6-diphosphate/myo-inositol diphosphates	1.41	3.129E-01	0.198
		dihydroxyacetone phosphate (DHAP)	0.66	5.011E-01	0.285
		3-phosphoglycerate	0.64	1.312E-01	0.099
		phosphoenolpyruvate (PEP)	**1.94**	6.100E-02	0.053
		pyruvate	**0.49**	8.908E-06	0.000
		lactate	0.93	4.810E-01	0.276
		glycerate	**1.66**	2.590E-02	0.026
	Pentose Phosphate Pathway	6-phosphogluconate	1.12	6.351E-01	0.337
		sedoheptulose-7-phosphate	**2.11**	8.100E-03	0.010
	Pentose Metabolism	ribose	**1.63**	2.290E-02	0.024
		ribitol	**0.44**	2.058E-06	0.000
		ribonate	**0.28**	2.668E-08	0.000
		ribulose/xylulose	**0.57**	1.900E-03	0.003
		arabinose	**0.64**	1.690E-02	0.018
		arabitol/xylitol	**0.66**	3.000E-03	0.004
		arabonate/xylonate	**0.67**	4.830E-02	0.044
		ribulonate/xylulonate/lyxonate*	0.91	5.735E-01	0.311
	Disaccharides and Oligosaccharides	lactose	**2.36**	4.300E-03	0.006
	Fructose, Mannose and Galactose Metabolism	fructose	1.62	1.577E-01	0.115
		mannitol/sorbitol	1.17	2.878E-01	0.186
		mannose	0.91	9.128E-01	0.433
		galactitol (dulcitol)	1.10	3.856E-01	0.233
		galactonate	**0.37**	6.797E-06	0.000
	Nucleotide Sugar	adenosine-5'-diphosphoglucose	1.00	1.000E+00	0.453
		UDP-glucose	**0.71**	1.540E-02	0.017
		UDP-galactose	0.87	2.402E-01	0.161
		UDP-glucuronate	**1.74**	4.782E-06	0.000
		guanosine 5'-diphospho-fucose	**1.19**	7.410E-02	0.062
		UDP-N-acetylglucosamine/galactosamine	**1.62**	3.800E-03	0.005
		cytidine 5'-monophospho-N-acetylneuraminic acid	**1.19**	9.160E-02	0.073
	Aminosugar Metabolism	glucosamine-6-phosphate	1.39	3.470E-01	0.214
		glucuronate	1.06	5.874E-01	0.316
		N-acetylglucosamine 6-phosphate	**3.88**	9.388E-07	0.000
		N-acetyl-glucosamine 1-phosphate	**0.42**	1.560E-05	0.000
		N-acetylneuraminate	**0.20**	1.663E-10	0.000
		N-acetylglucosaminylasparagine	1.10	1.633E-01	0.118
		erythronate*	**0.61**	2.000E-04	0.000
		N-acetylglucosamine/N-acetylgalactosamine	**1.38**	4.220E-02	0.040
		N-glycolylneuraminate	**0.29**	2.271E-07	0.000
	Advanced Glycation End-product	N6-carboxymethyllysine	1.25	3.388E-01	0.210
Energy	TCA Cycle	citrate	0.97	8.156E-01	0.401

		aconitate [cis or trans]	0.97	8.607E-01	0.417
		isocitrate	0.78	5.115E-01	0.288
		alpha-ketoglutarate	**0.50**	6.391E-05	0.000
		succinylcarnitine (C4-DC)	0.94	5.808E-01	0.314
		succinate	**0.86**	6.740E-02	0.057
		fumarate	**0.62**	8.000E-04	0.001
		malate	**0.60**	4.892E-05	0.000
		oxaloacetate	**0.54**	8.800E-03	0.010
		2-methylcitrate/homocitrate	1.14	3.210E-01	0.202
	Oxidative Phosphorylation	acetylphosphate	0.37	4.748E-01	0.274
		phosphate	0.94	4.168E-01	0.246
Lipid	Fatty Acid Synthesis	malonylcarnitine	1.07	5.732E-01	0.311
	Fatty Acid Metabolism	acetyl CoA	1.39	1.431E-01	0.107
		oleoyl CoA	0.83	5.022E-01	0.285
		arachidonoyl CoA	1.20	4.096E-01	0.244
	Short Chain Fatty Acid	butyrate/isobutyrate (4:0)	1.15	8.506E-01	0.415
	Medium Chain Fatty Acid	heptanoate (7:0)	0.95	6.118E-01	0.327
		(2 or 3)-decenoate (10:1n7 or n8)	0.73	2.534E-01	0.168
		5-dodecenoate (12:1n7)	0.81	4.886E-01	0.279
	Long Chain Saturated Fatty Acid	myristate (14:0)	0.88	6.567E-01	0.345
		pentadecanoate (15:0)	1.09	9.300E-01	0.436
		palmitate (16:0)	1.04	9.014E-01	0.428
		margarate (17:0)	1.74	6.412E-01	0.339
		stearate (18:0)	1.19	8.837E-01	0.425
		nonadecanoate (19:0)	1.67	6.630E-01	0.347
		arachidate (20:0)	1.40	8.705E-01	0.420
	Long Chain Monounsaturated Fatty Acid	myristoleate (14:1n5)	0.80	6.143E-01	0.327
		palmitoleate (16:1n7)	1.01	7.971E-01	0.394
		10-heptadecenoate (17:1n7)	2.29	5.463E-01	0.302
		oleate/vaccenate (18:1)	1.50	7.546E-01	0.381
		10-nonadecenoate (19:1n9)	2.08	5.591E-01	0.306
		eicosenoate (20:1)	1.24	9.468E-01	0.440
		erucate (22:1n9)	1.24	9.700E-01	0.445
	Long Chain Polyunsaturated Fatty Acid (n3 and n6)	eicosapentaenoate (EPA; 20:5n3)	7.71	1.698E-01	0.121
		heneicosapentaenoate (21:5n3)	**6.00**	8.550E-02	0.069
		docosapentaenoate (n3 DPA; 22:5n3)	6.82	1.369E-01	0.103
		docosahexaenoate (DHA; 22:6n3)	3.49	3.250E-01	0.204
		docosatrienoate (22:3n3)	2.37	6.166E-01	0.328
		nisinate (24:6n3)	2.25	5.518E-01	0.303
		hexadecadienoate (16:2n6)	1.61	5.112E-01	0.288
		linoleate (18:2n6)	2.46	4.573E-01	0.265
		linolenate [alpha or gamma; (18:3n3 or 6)]	1.41	6.969E-01	0.356
		dihomo-linoleate (20:2n6)	4.51	1.962E-01	0.136

		dihomo-linolenate (20:3n3 or n6)	3.18	2.404E-01	0.161
		arachidonate (20:4n6)	**8.11**	9.690E-02	0.076
		docosatrienoate (22:3n6)*	3.63	2.027E-01	0.139
		docosapentaenoate (n6 DPA; 22:5n6)	3.30	2.339E-01	0.157
		docosadienoate (22:2n6)	1.26	9.160E-01	0.433
		mead acid (20:3n9)	5.21	1.290E-01	0.098
	Fatty Acid, Branched	(12 or 13)-methylmyristate (a15:0 or i15:0)	1.07	9.402E-01	0.439
		(14 or 15)-methylpalmitate (a17:0 or i17:0)	1.26	9.816E-01	0.448
		(16 or 17)-methylstearate (a19:0 or i19:0)	1.21	9.358E-01	0.438
	Fatty Acid, Dicarboxylate	dimethylmalonic acid	1.54	1.644E-01	0.119
		glutarate (C5-DC)	1.09	6.824E-01	0.354
		2-hydroxyglutarate	**0.51**	5.000E-04	0.001
		2-hydroxyadipate	**1.67**	3.660E-02	0.036
		3-hydroxyadipate*	**1.42**	6.180E-02	0.053
		maleate	**0.72**	2.220E-02	0.023
		dodecadienoate (12:2)*	1.30	4.552E-01	0.264
	Fatty Acid Metabolism (also BCAA Metabolism)	butyrylcarnitine (C4)	**0.51**	1.000E-04	0.000
		propionylcarnitine (C3)	**0.57**	5.110E-05	0.000
		methylmalonate (MMA)	**1.87**	4.900E-03	0.007
	Fatty Acid Metabolism (Acyl Glycine)	N-palmitoylglycine	**0.28**	8.620E-02	0.070
	Fatty Acid Metabolism (Acyl Carnitine, Short Chain)	acetylcarnitine (C2)	**0.53**	3.000E-04	0.001
	Fatty Acid Metabolism (Acyl Carnitine, Medium Chain)	hexanoylcarnitine (C6)	**0.55**	1.600E-03	0.003
		octanoylcarnitine (C8)	**0.59**	2.550E-02	0.026
		decanoylcarnitine (C10)	0.74	3.327E-01	0.207
		laurylcarnitine (C12)	**0.46**	4.100E-03	0.006
	Fatty Acid Metabolism (Acyl Carnitine, Long Chain Saturated)	myristoylcarnitine (C14)	**0.26**	8.516E-06	0.000
		pentadecanoylcarnitine (C15)*	**0.56**	3.300E-02	0.032
		palmitoylcarnitine (C16)	**0.42**	1.300E-03	0.002
		margaroylcarnitine (C17)*	0.92	7.562E-01	0.381
		stearoylcarnitine (C18)	0.83	5.493E-01	0.302
		arachidoylcarnitine (C20)*	0.65	1.435E-01	0.107
	Fatty Acid Metabolism (Acyl Carnitine, Monounsaturated)	cis-4-decenoylcarnitine (C10:1)	0.77	2.603E-01	0.172
		5-dodecenoylcarnitine (C12:1)	**0.40**	2.000E-03	0.003
		myristoleoylcarnitine (C14:1)*	**0.52**	3.180E-02	0.032
		palmitoleoylcarnitine (C16:1)*	**0.30**	8.000E-04	0.002
		oleoylcarnitine (C18:1)	**0.44**	1.470E-02	0.016
		eicosenoylcarnitine (C20:1)*	**0.52**	3.180E-02	0.032
		erucoylcarnitine (C22:1)*	0.70	1.259E-01	0.096
	Fatty Acid Metabolism (Acyl Carnitine, Polyunsaturated)	linoleoylcarnitine (C18:2)*	**0.59**	6.280E-02	0.054
		linolenoylcarnitine (C18:3)*	**0.43**	4.900E-03	0.007
		dihomo-linoleoylcarnitine (C20:2)*	0.70	2.750E-01	0.180
		arachidonoylcarnitine (C20:4)	**0.58**	9.280E-02	0.074

		dihomo-linolenoylcarnitine (C20:3n3 or 6)*	**0.56**	7.710E-02	0.064
		adrenoylcarnitine (C22:4)*	0.72	3.670E-01	0.223
		docosapentaenoylcarnitine (C22:5n3)*	0.65	4.109E-01	0.244
		docosahexaenoylcarnitine (C22:6)*	**0.28**	2.250E-02	0.024
	Fatty Acid Metabolism (Acyl Carnitine, Hydroxy)	(R)-3-hydroxybutyrylcarnitine	**0.53**	4.188E-05	0.000
		(S)-3-hydroxybutyrylcarnitine	**0.36**	2.340E-06	0.000
		3-hydroxyhexanoylcarnitine (1)	1.33	1.428E-01	0.107
		3-hydroxydecanoylcarnitine	1.05	6.544E-01	0.345
		3-hydroxypalmitoylcarnitine	0.71	1.553E-01	0.114
		3-hydroxyoleoylcarnitine	0.87	4.331E-01	0.253
	Carnitine Metabolism	deoxycarnitine	**0.69**	5.200E-03	0.007
		carnitine	**0.47**	1.000E-04	0.000
	Ketone Bodies	3-hydroxybutyrate (BHBA)	**0.77**	5.240E-02	0.047
	Fatty Acid Metabolism (Acyl Choline)	palmitoylcholine	**5.15**	5.000E-04	0.001
		oleoylcholine	**3.86**	6.000E-04	0.001
		palmitoloelycholine	**2.66**	8.400E-03	0.010
		linoleoylcholine*	**1.90**	5.120E-02	0.046
		docosahexaenoylcholine	**6.46**	6.452E-07	0.000
		arachidonoylcholine	**1.52**	3.270E-02	0.032
	Fatty Acid, Monohydroxy	4-hydroxybutyrate (GHB)	**1.74**	1.400E-03	0.002
		2-hydroxypalmitate	1.34	9.702E-01	0.445
		2-hydroxystearate	1.38	9.634E-01	0.444
		3-hydroxyhexanoate	1.31	4.003E-01	0.239
		3-hydroxyoctanoate	0.95	9.141E-01	0.433
		3-hydroxydecanoate	1.05	6.838E-01	0.354
		3-hydroxytridecanoate	1.07	9.162E-01	0.433
		3-hydroxylaurate	1.04	7.625E-01	0.382
		3-hydroxymyristate	0.90	7.395E-01	0.375
		3-hydroxypalmitate	0.98	7.395E-01	0.375
		3-hydroxystearate	1.08	8.621E-01	0.417
		3-hydroxyoleate*	1.16	8.267E-01	0.405
		9-hydroxystearate	**0.24**	5.220E-02	0.047
	Fatty Acid, Dihydroxy	2S,3R-dihydroxybutyrate	0.96	8.701E-01	0.420
		2R,3R-dihydroxybutyrate	**0.58**	7.800E-03	0.010
		2,4-dihydroxybutyrate	0.98	9.765E-01	0.446
	Endocannabinoid	oleoyl ethanolamide	**2.15**	5.000E-04	0.001
		palmitoyl ethanolamide	**1.37**	6.740E-02	0.057
		stearoyl ethanolamide	**2.15**	6.000E-04	0.001
		arachidonoyl ethanolamide	**2.95**	2.970E-02	0.030
		N-myristoyltaurine*	**4.27**	1.840E-02	0.020
		N-arachidonoyltaurine	**10.42**	4.520E-02	0.042
		N-oleoyltaurine	**7.72**	5.150E-02	0.046
		N-stearoyltaurine	5.27	1.054E-01	0.082

		N-palmitoyltaurine	**6.33**	9.480E-02	0.075
		N-linoleoyltaurine*	**10.28**	3.960E-02	0.038
		linoleoyl ethanolamide	1.62	8.903E-01	0.427
		palmitoleoyl ethanolamide*	0.87	6.583E-01	0.345
		N-oleoylserine	1.64	5.583E-01	0.306
	Inositol Metabolism	myo-inositol	**0.48**	8.017E-07	0.000
		inositol 1-phosphate (I1P)	**2.32**	3.000E-04	0.001
	Phospholipid Metabolism	choline	**1.19**	2.740E-02	0.028
		choline phosphate	**1.69**	1.000E-03	0.002
		cytidine 5'-diphosphocholine	**1.56**	1.000E-04	0.000
		glycerophosphorylcholine (GPC)	**4.29**	4.159E-06	0.000
		phosphoethanolamine	**10.38**	5.707E-08	0.000
		cytidine-5'-diphosphoethanolamine	**2.02**	2.050E-06	0.000
		glycerophosphoethanolamine	**3.13**	6.434E-07	0.000
		glycerophosphoserine*	**0.81**	1.180E-02	0.014
		glycerophosphoinositol*	**0.82**	4.760E-02	0.044
		trimethylamine N-oxide	**0.54**	1.390E-06	0.000
	Phosphatidylcholine (PC)	1-myristoyl-2-palmitoyl-GPC (14:0/16:0)	0.87	2.250E-01	0.153
		1-myristoyl-2-arachidonoyl-GPC (14:0/20:4)*	**2.89**	5.208E-05	0.000
		1,2-dipalmitoyl-GPC (16:0/16:0)	0.85	2.309E-01	0.155
		1-palmitoyl-2-palmitoleoyl-GPC (16:0/16:1)*	**0.79**	6.420E-02	0.055
		1-palmitoyl-2-stearoyl-GPC (16:0/18:0)	1.07	6.751E-01	0.351
		1-palmitoyl-2-oleoyl-GPC (16:0/18:1)	1.03	7.647E-01	0.383
		1-palmitoyl-2-arachidonoyl-GPC (16:0/20:4n6)	**2.90**	5.381E-05	0.000
		1-palmitoyl-2-docosahexaenoyl-GPC (16:0/22:6)	**2.86**	2.000E-04	0.000
		1-palmitoleoyl-2-linolenoyl-GPC (16:1/18:3)*	**0.66**	3.800E-03	0.005
		1-stearoyl-2-oleoyl-GPC (18:0/18:1)	1.20	1.796E-01	0.127
		1-stearoyl-2-linoleoyl-GPC (18:0/18:2)*	1.00	9.715E-01	0.445
		1-stearoyl-2-arachidonoyl-GPC (18:0/20:4)	**3.32**	1.431E-05	0.000
		1-stearoyl-2-docosahexaenoyl-GPC (18:0/22:6)	**3.07**	3.005E-05	0.000
		1,2-dioleoyl-GPC (18:1/18:1)	0.81	1.467E-01	0.109
		1-oleoyl-2-docosahexaenoyl-GPC (18:1/22:6)*	**1.99**	1.600E-03	0.003
		1,2-dilinoleoyl-GPC (18:2/18:2)	1.30	1.749E-01	0.125
	Phosphatidylethanolamine (PE)	1,2-dipalmitoyl-GPE (16:0/16:0)*	**0.74**	5.130E-02	0.046
		1-palmitoyl-2-oleoyl-GPE (16:0/18:1)	**0.76**	4.910E-02	0.045
		1-palmitoyl-2-arachidonoyl-GPE (16:0/20:4)*	1.23	1.660E-01	0.119
		1-palmitoyl-2-docosahexaenoyl-GPE (16:0/22:6)*	**2.08**	4.000E-04	0.001
		1-stearoyl-2-oleoyl-GPE (18:0/18:1)	1.02	9.223E-01	0.433
		1-stearoyl-2-arachidonoyl-GPE (18:0/20:4)	**1.27**	7.780E-02	0.064
		1-oleoyl-2-linoleoyl-GPE (18:1/18:2)*	1.08	5.434E-01	0.302

		1-oleoyl-2-arachidonoyl-GPE (18:1/20:4)*	**1.63**	6.600E-03	0.008
		1-oleoyl-2-docosahexaenoyl-GPE (18:1/22:6)*	**1.83**	7.000E-04	0.001
	Phosphatidylserine (PS)	1-palmitoyl-2-oleoyl-GPS (16:0/18:1)	**0.63**	9.900E-03	0.012
		1-stearoyl-2-oleoyl-GPS (18:0/18:1)	0.83	2.274E-01	0.154
		1-stearoyl-2-arachidonoyl-GPS (18:0/20:4)	**1.60**	2.560E-02	0.026
	Phosphatidylglycerol (PG)	1-palmitoyl-2-oleoyl-GPG (16:0/18:1)	1.16	5.663E-01	0.309
	Phosphatidylinositol (PI)	1-palmitoyl-2-oleoyl-GPI (16:0/18:1)*	0.98	9.375E-01	0.438
		1-palmitoyl-2-arachidonoyl-GPI (16:0/20:4)*	**1.66**	4.640E-02	0.043
		1-stearoyl-2-oleoyl-GPI (18:0/18:1)*	0.91	7.823E-01	0.389
		1-stearoyl-2-arachidonoyl-GPI (18:0/20:4)	1.24	6.699E-01	0.349
		1-oleoyl-2-arachidonoyl-GPI (18:1/20:4)*	**1.46**	9.110E-02	0.073
	Lysophospholipid	1-palmitoyl-GPC (16:0)	1.23	2.739E-01	0.179
		2-palmitoyl-GPC (16:0)*	1.16	9.415E-01	0.439
		1-palmitoleoyl-GPC (16:1)*	0.91	4.396E-01	0.257
		2-palmitoleoyl-GPC (16:1)*	**0.78**	5.300E-02	0.047
		1-stearoyl-GPC (18:0)	**1.95**	1.900E-03	0.003
		1-oleoyl-GPC (18:1)	1.07	7.994E-01	0.395
		1-lignoceroyl-GPC (24:0)	1.08	6.935E-01	0.356
		1-palmitoyl-GPE (16:0)	1.47	1.090E-01	0.084
		1-stearoyl-GPE (18:0)	**1.95**	2.200E-03	0.003
		2-stearoyl-GPE (18:0)*	0.97	9.602E-01	0.443
		1-oleoyl-GPE (18:1)	**1.50**	1.300E-03	0.002
		1-linoleoyl-GPE (18:2)*	**1.37**	9.500E-03	0.011
		1-arachidonoyl-GPE (20:4n6)*	**1.42**	1.200E-03	0.002
		1-palmitoyl-GPS (16:0)*	1.86	5.476E-01	0.302
		1-stearoyl-GPS (18:0)*	**2.58**	1.300E-03	0.002
		1-oleoyl-GPS (18:1)	1.63	5.646E-01	0.309
		1-palmitoyl-GPG (16:0)*	0.78	5.480E-01	0.302
		1-stearoyl-GPG (18:0)	1.04	8.952E-01	0.428
		1-oleoyl-GPG (18:1)*	1.03	7.724E-01	0.385
		1-palmitoyl-GPI (16:0)	2.24	4.138E-01	0.245
		1-stearoyl-GPI (18:0)	1.63	6.295E-01	0.334
		1-oleoyl-GPI (18:1)	**1.98**	4.830E-02	0.044
		1-arachidonoyl-GPI (20:4)*	2.20	3.467E-01	0.214
	Plasmalogen	1-(1-enyl-palmitoyl)-2-oleoyl-GPE (P-16:0/18:1)*	0.88	3.003E-01	0.193
		1-(1-enyl-palmitoyl)-2-linoleoyl-GPE (P-16:0/18:2)*	0.93	5.335E-01	0.299
		1-(1-enyl-palmitoyl)-2-palmitoyl-GPC (P-16:0/16:0)*	**1.34**	7.580E-02	0.063
		1-(1-enyl-palmitoyl)-2-palmitoleoyl-GPC (P-16:0/16:1)*	1.06	6.958E-01	0.356
		1-(1-enyl-palmitoyl)-2-arachidonoyl-GPE (P-16:0/20:4)*	**1.35**	1.900E-02	0.020
		1-(1-enyl-palmitoyl)-2-oleoyl-GPC (P-16:0/18:1)*	**1.29**	9.970E-02	0.078

		1-(1-enyl-stearoyl)-2-oleoyl-GPE (P-18:0/18:1)	0.88	4.872E-01	0.279
		1-(1-enyl-palmitoyl)-2-arachidonoyl-GPC (P-16:0/20:4)*	**5.25**	6.756E-07	0.000
		1-(1-enyl-palmitoyl)-2-linoleoyl-GPC (P-16:0/18:2)*	**1.79**	6.600E-03	0.008
		1-(1-enyl-stearoyl)-2-arachidonoyl-GPE (P-18:0/20:4)*	**1.59**	3.100E-03	0.005
	Lysoplasmalogen	1-(1-enyl-palmitoyl)-GPC (P-16:0)*	**3.43**	1.440E-05	0.000
		1-(1-enyl-palmitoyl)-GPE (P-16:0)*	**2.35**	9.000E-04	0.002
		1-(1-enyl-oleoyl)-GPE (P-18:1)*	**2.36**	1.000E-03	0.002
		1-(1-enyl-stearoyl)-GPE (P-18:0)*	**2.83**	6.281E-05	0.000
	Glycerolipid Metabolism	glycerol	0.90	2.895E-01	0.187
		glycerol 3-phosphate	**1.86**	2.590E-02	0.026
		glycerophosphoglycerol	0.89	2.651E-01	0.174
	Monoacylglycerol	1-myristoylglycerol (14:0)	0.69	4.992E-01	0.284
		1-pentadecanoylglycerol (15:0)	1.13	9.427E-01	0.439
		1-palmitoylglycerol (16:0)	0.83	5.305E-01	0.297
		1-palmitoleoylglycerol (16:1)*	0.57	3.258E-01	0.204
		1-margaroylglycerol (17:0)	1.22	8.985E-01	0.428
		1-oleoylglycerol (18:1)	0.95	6.941E-01	0.356
		1-linoleoylglycerol (18:2)	1.31	9.677E-01	0.445
		1-dihomo-linolenylglycerol (20:3)	1.57	9.893E-01	0.451
		1-arachidonylglycerol (20:4)	2.17	4.427E-01	0.258
		1-docosahexaenoylglycerol (22:6)	1.45	9.923E-01	0.452
		2-myristoylglycerol (14:0)	0.72	5.479E-01	0.302
		2-palmitoylglycerol (16:0)	0.95	7.658E-01	0.383
		2-palmitoleoylglycerol (16:1)*	0.65	5.071E-01	0.287
		2-oleoylglycerol (18:1)	1.07	8.570E-01	0.417
		2-linoleoylglycerol (18:2)	1.80	7.405E-01	0.375
		2-arachidonoylglycerol (20:4)	2.43	5.460E-01	0.302
		2-docosahexaenoylglycerol (22:6)*	1.75	7.586E-01	0.381
		1-heptadecenoylglycerol (17:1)*	0.97	7.726E-01	0.385
		2-heptadecenoylglycerol (17:1)*	1.42	8.861E-01	0.426
	Diacylglycerol	palmitoyl-oleoyl-glycerol (16:0/18:1) [2]*	**0.38**	4.000E-04	0.001
		oleoyl-arachidonoyl-glycerol (18:1/20:4) [2]*	1.33	6.146E-01	0.327
	Galactosyl Glycerolipids	galactosylglycerol	**0.65**	1.200E-03	0.002
	Sphingolipid Synthesis	3-ketosphinganine	**5.91**	2.306E-10	0.000
		sphinganine	**2.35**	5.000E-04	0.001
		sphingadienine	**3.00**	3.400E-03	0.005
		phytosphingosine	1.17	2.284E-01	0.155
	Dihydroceramides	N-palmitoyl-sphinganine (d18:0/16:0)	**3.08**	1.170E-02	0.014
		N-stearoyl-sphinganine (d18:0/18:0)*	**3.64**	1.420E-02	0.016
	Ceramides	N-palmitoyl-sphingosine (d18:1/16:0)	**2.33**	4.800E-03	0.007
		N-stearoyl-sphingosine (d18:1/18:0)*	**2.04**	8.600E-03	0.010

		N-palmitoyl-sphingadienine (d18:2/16:0)*	**1.87**	6.600E-03	0.008
		ceramide (d18:1/14:0, d16:1/16:0)*	**1.79**	1.420E-02	0.016
		ceramide (d18:1/17:0, d17:1/18:0)*	**3.31**	2.400E-03	0.004
		ceramide (d16:1/24:1, d18:1/22:1)*	1.88	5.181E-01	0.291
		ceramide (d18:2/24:1, d18:1/24:2)*	1.34	2.250E-01	0.153
	Hexosylceramides (HCER)	glycosyl-N-stearoyl-sphinganine (d18:0/18:0)*	**5.23**	2.300E-03	0.003
		glycosyl-N-palmitoyl-sphingosine (d18:1/16:0)	1.32	1.955E-01	0.136
		glycosyl-N-stearoyl-sphingosine (d18:1/18:0)	1.51	1.098E-01	0.084
		glycosyl-N-behenoyl-sphingadienine (d18:2/22:0)*	1.36	2.785E-01	0.182
		glycosyl ceramide (d18:1/20:0, d16:1/22:0)*	**2.13**	7.000E-03	0.009
		glycosyl ceramide (d16:1/24:1, d18:1/22:1)*	**2.52**	8.600E-03	0.010
		glycosyl ceramide (d18:1/23:1, d17:1/24:1)*	**1.67**	7.280E-02	0.061
		glycosyl ceramide (d18:2/24:1, d18:1/24:2)*	1.08	8.017E-01	0.395
	Lactosylceramides (LCER)	lactosyl-N-palmitoyl-sphingosine (d18:1/16:0)	**1.92**	2.000E-04	0.001
		lactosyl-N-stearoyl-sphingosine (d18:1/18:0)*	**2.07**	8.000E-04	0.002
		lactosyl-N-behenoyl-sphingosine (d18:1/22:0)*	**1.79**	1.590E-02	0.017
		lactosyl-N-nervonoyl-sphingosine (d18:1/24:1)*	**1.79**	2.500E-03	0.004
	Dihydrosphingomyelins	myristoyl dihydrosphingomyelin (d18:0/14:0)*	**1.59**	3.720E-02	0.036
		palmitoyl dihydrosphingomyelin (d18:0/16:0)*	**1.81**	1.470E-02	0.016
		behenoyl dihydrosphingomyelin (d18:0/22:0)*	**2.16**	3.950E-02	0.038
		sphingomyelin (d18:0/18:0, d19:0/17:0)*	**2.90**	1.800E-03	0.003
		sphingomyelin (d18:0/20:0, d16:0/22:0)*	**2.67**	1.900E-03	0.003
	Sphingomyelins	palmitoyl sphingomyelin (d18:1/16:0)	**1.37**	2.330E-02	0.024
		stearoyl sphingomyelin (d18:1/18:0)	**1.42**	6.300E-03	0.008
		behenoyl sphingomyelin (d18:1/22:0)*	**1.90**	2.610E-02	0.026
		tricosanoyl sphingomyelin (d18:1/23:0)*	1.31	2.634E-01	0.173
		lignoceroyl sphingomyelin (d18:1/24:0)	1.42	1.973E-01	0.137
		sphingomyelin (d18:2/23:1)*	1.26	2.817E-01	0.183
		sphingomyelin (d18:2/24:2)*	0.91	6.481E-01	0.342
		sphingomyelin (d17:1/14:0, d16:1/15:0)*	1.10	6.643E-01	0.347
		sphingomyelin (d18:1/14:0, d16:1/16:0)*	1.28	1.349E-01	0.102
		sphingomyelin (d18:2/14:0, d18:1/14:1)*	0.81	1.899E-01	0.134
		sphingomyelin (d17:1/16:0, d18:1/15:0, d16:1/17:0)*	1.29	1.647E-01	0.119
		sphingomyelin (d17:2/16:0, d18:2/15:0)*	1.24	3.289E-01	0.205
		sphingomyelin (d18:2/16:0, d18:1/16:1)*	1.17	3.269E-01	0.205
		sphingomyelin (d18:1/17:0, d17:1/18:0, d19:1/16:0)	**1.92**	2.200E-03	0.003
		sphingomyelin (d18:1/18:1, d18:2/18:0)	1.10	4.098E-01	0.244
		sphingomyelin (d18:1/20:0, d16:1/22:0)*	**1.76**	3.900E-03	0.005

		sphingomyelin (d18:1/21:0, d17:1/22:0, d16:1/23:0)*	**2.03**	1.290E-02	0.015
		sphingomyelin (d18:1/22:1, d18:2/22:0, d16:1/24:1)*	**1.37**	9.860E-02	0.077
		sphingomyelin (d18:1/22:2, d18:2/22:1, d16:1/24:2)*	1.50	4.958E-01	0.283
		sphingomyelin (d18:2/23:0, d18:1/23:1, d17:1/24:1)*	1.38	1.925E-01	0.135
		sphingomyelin (d18:1/24:1, d18:2/24:0)*	1.23	3.718E-01	0.225
		sphingomyelin (d18:2/24:1, d18:1/24:2)*	0.98	8.964E-01	0.428
	Sphingosines	sphingosine	**1.97**	1.200E-02	0.014
		sphingosine 1-phosphate	**1.58**	4.440E-02	0.042
		hexadecasphingosine (d16:1)*	**3.35**	1.200E-03	0.002
		heptadecasphingosine (d17:1)	**2.11**	1.660E-02	0.018
		eicosanoylsphingosine (d20:1)*	**4.11**	2.000E-04	0.001
	Mevalonate Metabolism	3-hydroxy-3-methylglutarate	**0.34**	2.425E-09	0.000
	Sterol	cholesterol	0.95	5.815E-01	0.314
		7-dehydrocholesterol	**3.44**	7.249E-07	0.000
		4-cholesten-3-one	1.27	4.720E-01	0.272
		beta-sitosterol	**1.70**	1.500E-02	0.017
		campesterol	1.59	1.948E-01	0.136
		7-hydroxycholesterol (alpha or beta)	**1.61**	1.840E-02	0.020
	Primary Bile Acid Metabolism	glycochenodeoxycholate	1.62	1.879E-01	0.133
		taurochenodeoxycholate	1.64	1.439E-01	0.107
	Secondary Bile Acid Metabolism	glycodeoxycholate	1.30	6.860E-01	0.354
Nucleotide	Purine Metabolism, (Hypo)Xanthine/Inosine containing)	AICA ribonucleotide	**0.17**	4.685E-05	0.000
		inosine 5'-monophosphate (IMP)	**0.44**	9.070E-02	0.073
		inosine	0.85	5.107E-01	0.288
		hypoxanthine	0.82	3.632E-01	0.221
		xanthine	1.02	7.842E-01	0.389
		xanthosine	**1.56**	6.010E-02	0.052
		N1-methylinosine	**2.81**	6.000E-04	0.001
		2'-deoxyinosine	**0.12**	3.792E-06	0.000
		urate	**0.48**	1.100E-03	0.002
		allantoin	**0.80**	1.130E-02	0.013
	Purine Metabolism, Adenine containing	adenosine 5'-triphosphate (ATP)	0.95	9.180E-01	0.433
		adenosine 5'-diphosphate (ADP)	1.00	8.198E-01	0.402
		adenosine 5'-monophosphate (AMP)	0.96	8.753E-01	0.422
		adenosine 3',5'-cyclic monophosphate (cAMP)	**0.72**	2.200E-03	0.003
		adenylosuccinate	**0.51**	5.200E-03	0.007
		adenosine	1.04	5.939E-01	0.319
		adenine	**0.71**	3.070E-02	0.031
		N1-methyladenosine	**1.86**	1.224E-05	0.000
		N6-methyladenosine	0.86	6.713E-01	0.349
		N6-carbamoylthreonyladenosine	**1.15**	8.380E-02	0.068

		2'-deoxyadenosine 5'-diphosphate	**0.58**	7.700E-03	0.010
		2'-deoxyadenosine 5'-monophosphate	**0.04**	6.235E-12	0.000
		2'-deoxyadenosine	**0.24**	2.000E-04	0.000
		diadenosine triphosphate	0.93	5.821E-01	0.314
		N6-succinyladenosine	1.16	3.533E-01	0.217
	Purine Metabolism, Guanine containing	guanosine 5'- diphosphate (GDP)	0.94	8.059E-01	0.397
		guanosine 5'- monophosphate (5'-GMP)	**0.78**	5.680E-02	0.050
		guanosine	0.76	1.927E-01	0.135
		guanine	1.39	1.185E-01	0.091
		7-methylguanine	**0.60**	6.049E-05	0.000
		N2-methylguanosine	1.21	1.059E-01	0.082
		N2,N2-dimethylguanosine	**0.83**	8.090E-02	0.066
		2'-deoxyguanosine	**0.25**	2.427E-05	0.000
	Pyrimidine Metabolism, Orotate containing	dihydroorotate	**0.21**	9.000E-03	0.011
		orotate	**0.14**	3.000E-04	0.001
		orotidine	**0.02**	4.900E-14	0.000
	Pyrimidine Metabolism, Uracil containing	uridine 5'-triphosphate (UTP)	1.09	6.865E-01	0.354
		uridine 5'-diphosphate (UDP)	0.79	3.963E-01	0.238
		uridine 5'-monophosphate (UMP)	0.84	3.901E-01	0.235
		uridine 3'-monophosphate (3'-UMP)	**2.99**	6.800E-03	0.009
		uridine	**0.71**	1.540E-02	0.017
		uracil	0.67	1.892E-01	0.134
		pseudouridine	1.08	3.396E-01	0.210
		5,6-dihydrouridine	**1.36**	8.000E-04	0.001
		2'-O-methyluridine	1.11	3.174E-01	0.201
		5-methyluridine (ribothymidine)	**0.22**	1.196E-05	0.000
		2'-deoxyuridine	0.63	1.521E-01	0.112
		3-ureidopropionate	**0.22**	1.211E-09	0.000
		beta-alanine	**0.35**	1.419E-06	0.000
		3-(3-amino-3-carboxypropyl)uridine*	**0.59**	1.066E-05	0.000
	Pyrimidine Metabolism, Cytidine containing	cytidine triphosphate	1.69	2.915E-01	0.188
		cytidine diphosphate	**1.47**	7.830E-02	0.064
		cytidine 5'-monophosphate (5'-CMP)	**1.19**	6.650E-02	0.057
		cytidine	**1.30**	7.000E-02	0.059
		cytosine	**1.54**	2.770E-02	0.028
		3-methylcytidine	**1.86**	2.751E-05	0.000
		5-methylcytidine	**0.50**	9.964E-07	0.000
		2'-deoxycytidine 5'-monophosphate	**0.73**	4.660E-02	0.043
		2'-deoxycytidine	0.80	7.086E-01	0.362
		2'-O-methylcytidine	**1.94**	9.866E-06	0.000
	Pyrimidine Metabolism, Thymine containing	thymidine 5'-monophosphate	0.99	9.003E-01	0.428
		thymidine	1.31	1.510E-01	0.112
		thymine	**0.59**	1.450E-02	0.016

		5,6-dihydrothymine	0.91	6.200E-03	0.008
		3-aminoisobutyrate	0.74	1.125E-01	0.086
	Purine and Pyrimidine Metabolism	methylphosphate	1.51	3.270E-02	0.032
Cofactors and Vitamins	Nicotinate and Nicotinamide Metabolism	quinolinate	1.13	7.972E-01	0.394
		nicotinamide	0.96	9.494E-01	0.440
		nicotinamide ribonucleotide (NMN)	0.64	4.970E-02	0.045
		nicotinamide riboside	1.17	1.525E-01	0.112
		nicotinamide adenine dinucleotide (NAD+)	0.77	4.400E-03	0.006
		nicotinamide adenine dinucleotide reduced (NADH)	0.57	1.650E-02	0.018
		nicotinamide adenine dinucleotide phosphate reduced (NADPH)	0.66	3.726E-01	0.225
		1-methylnicotinamide	1.33	2.030E-02	0.021
		trigonelline (N'-methylnicotinate)	0.73	5.680E-02	0.050
		adenosine 5'-diphosphoribose (ADP-ribose)	0.95	8.581E-01	0.417
	Riboflavin Metabolism	riboflavin (Vitamin B2)	1.42	2.000E-04	0.000
		flavin adenine dinucleotide (FAD)	1.02	6.927E-01	0.356
		flavin mononucleotide (FMN)	0.92	4.302E-01	0.252
	Pantothenate and CoA Metabolism	pantoate	0.45	3.477E-05	0.000
		pantothenate	0.92	3.631E-01	0.221
		pantetheine	1.59	2.900E-03	0.004
		phosphopantetheine	1.76	1.420E-02	0.016
		3'-dephosphocoenzyme A	2.31	3.900E-03	0.005
		coenzyme A	1.15	2.997E-01	0.193
	Ascorbate and Aldarate Metabolism	2-O-methylascorbic acid	1.00	9.660E-01	0.445
		threonate	1.00	1.000E+00	0.453
		gulonate*	0.54	4.711E-05	0.000
	Tocopherol Metabolism	alpha-tocopherol	1.04	7.237E-01	0.369
	Biotin Metabolism	biotin	0.84	5.854E-01	0.315
	Folate Metabolism	folate	1.00	1.000E+00	0.453
		5-methyltetrahydrofolate (5MeTHF)	0.49	1.000E-04	0.000
	Pterin Metabolism	pterin	0.73	3.395E-01	0.210
	Hemoglobin and Porphyrin Metabolism	bilirubin (Z,Z)	0.75	1.338E-01	0.101
	Thiamine Metabolism	thiamin (Vitamin B1)	2.83	1.975E-06	0.000
		thiamin monophosphate	1.50	1.782E-01	0.127
		thiamin diphosphate	1.50	1.073E-01	0.083
		5-(2-Hydroxyethyl)-4-methylthiazole	2.57	2.201E-05	0.000
	Vitamin A Metabolism	retinol (Vitamin A)	1.73	7.000E-04	0.001
	Vitamin B6 Metabolism	pyridoxine (Vitamin B6)	0.04	8.950E-13	0.000
		pyridoxamine	0.66	8.100E-03	0.010
		pyridoxamine phosphate	0.89	3.036E-01	0.194
		pyridoxal phosphate	0.68	2.000E-04	0.001
		pyridoxal	0.56	4.753E-05	0.000
		pyridoxate	4.53	5.221E-08	0.000

Xenobiotics	Benzoate Metabolism	hippurate	1.36	3.515E-01	0.216
		3-hydroxyhippurate	1.53	2.627E-01	0.173
		benzoate	0.70	1.609E-01	0.117
		catechol sulfate	1.60	1.350E-01	0.102
		guaiacol sulfate	**1.57**	3.800E-02	0.037
		4-methylcatechol sulfate	1.33	1.559E-01	0.114
		p-cresol sulfate	**1.68**	1.000E-04	0.000
	Food Component/Plant	3-formylindole	**1.44**	4.230E-02	0.040
		gluconate	0.59	3.388E-01	0.210
		beta-guanidinopropanoate	**0.53**	1.400E-03	0.002
		ergothioneine	**0.61**	1.040E-02	0.012
		erythritol	**0.82**	6.600E-03	0.008
		homostachydrine*	**0.50**	7.727E-07	0.000
		mannonate*	**0.42**	1.754E-06	0.000
		stachydrine	**0.47**	6.798E-07	0.000
		methyl glucopyranoside (alpha + beta)	**3.31**	1.933E-06	0.000
		ethyl beta-glucopyranoside	0.35	4.159E-01	0.246
		2-aminophenol sulfate	**0.46**	8.000E-04	0.001
	Drug - Antibiotic	penicillin G	1.15	5.355E-01	0.299
	Chemical	sulfate*	0.84	7.547E-01	0.381
		O-sulfo-L-tyrosine	1.10	2.825E-01	0.183
		2,4-di-tert-butylphenol	0.79	9.538E-01	0.441
		phenol red	1.17	3.055E-01	0.195
		thioproline	1.01	9.527E-01	0.441
		4-chlorobenzoic acid	0.97	8.919E-01	0.427
Partially Characterized Molecules	Partially Characterized Molecules	branched-chain, straight-chain, or cyclopropyl 12:1 fatty acid*	1.04	9.218E-01	0.433
